# Systemic Defenses to Prevent Intravenous Medication Errors in Hospitals: A Systematic Review

**DOI:** 10.1097/PTS.0000000000000688

**Published:** 2020-03-17

**Authors:** Sini Karoliina Kuitunen, Ilona Niittynen, Marja Airaksinen, Anna-Riia Holmström

**Affiliations:** From the ∗ HUS Pharmacy, Hospital Pharmacy of Helsinki University Hospital (HUS); † Clinical Pharmacy Group, Faculty of Pharmacy, University of Helsinki; Helsinki, Finland.

**Keywords:** patient safety, medication safety, intravenous medication, medication error, systemic defense, risk management, systematic review

## Abstract

**Objectives:**

Intravenous medication delivery is a complex process that poses systemic risks of errors. The objective of our study was to identify systemic defenses that can prevent in-hospital intravenous (IV) medication errors.

**Methods:**

A systematic review adhering to Preferred Reporting Items for Systematic Reviews and Meta-Analyses guidelines was conducted. We searched MEDLINE (Ovid), Scopus, CINAHL, and EMB reviews for articles published between January 2005 and June 2016. Peer-reviewed journal articles published in English were included. Two reviewers independently selected articles according to a predetermined PICO tool. The quality of studies was assessed using the Grading of Recommendations Assessment, Development and Evaluation system, and the evidence was analyzed using qualitative content analysis.

**Results:**

Forty-six studies from 11 countries were included in the analysis. We identified systemic defenses related to administration (n = 24 studies), prescribing (n = 8), preparation (n = 6), treatment monitoring (n = 2), and dispensing (n = 1). In addition, 5 studies explored defenses related to multiple stages of the drug delivery process. Systemic defenses including features of closed-loop medication management systems appeared in 61% of the studies, with smart pumps being the defense most widely studied (24%). The evidence quality of the included articles was limited, as 83% were graded as low quality, 13% were of moderate quality, and only 4% were of high quality.

**Conclusions:**

In-hospital IV medication processes are developing toward closed-loop medication management systems. Our study provides health care organizations with preliminary knowledge about systemic defenses that can prevent IV medication errors, but more rigorous evidence is needed. There is a need for further studies to explore combinations of different systemic defenses and their effectiveness in error prevention throughout the drug delivery process.

## Abstract 2

Supplemental digital content is available in the text.

Intravenous drug delivery is a complex process involving multiple possibilities for error.^1,2^ Because of the immediate therapeutic effect and high bioavailability, intravenous (IV) administration routes are widely used in hospitals and especially in critical care settings, such as intensive care units and emergency departments. However, many intravenously administered drugs are high-alert medications, bearing a heightened risk of causing significant patient harm if used in error.^3^ Intravenously administered drugs are associated with the highest medication error frequencies and more serious consequences to the patient than any other administration route.^4–6^ A meta-analysis of observational studies from the United Kingdom demonstrated that administration errors are as much as 5 times more likely when an IV route is used.^7^ Recent observational multisite studies conducted in the United States and the United Kingdom have reported a high prevalence of IV infusion administration errors and procedural failures, even with the use of smart pumps, yet few potentially harmful errors.^8,9^

To ensure medication safety, effective interventions that can eliminate errors in the IV drug delivery process are needed. In health care, the framework of a just culture ensures balanced accountability for both individuals and the organization responsible for designing and improving systems in the workplace.^10^ From an organizational point of view, it is essential to identify weaknesses of the current practices and develop systemic defenses to prevent errors reaching patients.^11,12^ Currently, many systemic defenses involve technology-based solutions, and in-hospital drug delivery processes have developed toward closed-loop medication management. Closed-loop systems consist of electronic prescribing, dispensing of bar-coded unit-dose drugs, safe storage in automated dispensing cabinets, barcode scanning to confirm drug and patient identity, electronic administration records, and clinical decision systems supporting every process step from prescribing to treatment monitoring.^13–17^ Although most IV medication errors happen in the administration stage,^6,18,19^ smart infusion pumps using dose error reduction software are an essential part of IV closed-loop systems.^20^ However, other types of systemic defenses are also needed to ensure IV medication safety, such as the use of oral syringes that do not fit to IV lines to prevent inadvertent IV administration of oral solutions.^21^

To the best of our knowledge, the systemic defenses related to IV medication processes have not been systematically reviewed before. Previous systematic reviews have focused on error prevention strategies in general (e.g., interventions to reduce medication errors in adult^22^ and pediatric^23^ intensive care) or one systemic defense (e.g., smart infusion pumps^24^). Although medication safety is a global priority, systemic defenses related to certain administration routes are not clearly described in many countries. The aim of our study was to explore recent evidence of systemic defenses and their ability to prevent IV medication errors to inform interprofessional medication safety activities in hospitals.

## METHODS

### Study Design

A systematic review of recent evidence on systemic defenses aiming to prevent IV medication errors in hospitals was carried out following the Preferred Reporting Items for Systematic Reviews and Meta-Analyses guidelines (PRISMA) for undertaking and presenting systematic reviews.^25^ The quality of the included studies was assessed according to the Grading of Recommendations Assessment, Development and Evaluation (GRADE) system.^26^ The included articles were analyzed using qualitative content analysis.^27,28^

### Search Strategy

A systematic literature search was performed in June 2016 on MEDLINE (Ovid), Scopus, CINAHL, and EMB reviews covering the period from January 2005 to June 2016. This period was chosen to focus on the most recent evidence published in peer-reviewed journals. An example of the search strategy is presented in Table 1.

**TABLE 1:** Search Strategy for MEDLINE (Ovid)

1. Infusions, intravenous/ or injections, intravenous/
2. Intravenous*
3. Infusion* adj3 drip*
4. 1 or 2 or 3
5. Medication errors/
6. Medication* adj3 error*
7. Administration* adj3 error*
8. Prescribing* adj3 error*
9. Dispensing* adj3 error*
10. Drug* adj3 error*
11. Drug* adj3 mistake*
12. Drug* adj3 mishap*
13. Medication* adj3 mistake*
14. Medication* adj3 mishap*
15. Administration* adj3 mistake*
16. Dispensing* adj3 mistake*
17. Prescribing* adj3 mistake*
18. Wrong* adj3 drug*
19. Wrong* adj3 dose*
20. Incorrect* adj3 drug*
21. Incorrect* adj3 dose*
22. Incorrect* adj3 administration* adj3 route*
23. Drug* adj3 death*
24. Medication* adj3 safety*
25. Medication* adj3 event*
26. Medication* adj3 incident*
27. 5 or 6 or 7 or 8 or 9 or 10 or 11 or 12 or 13 or 14 or 15 or 16 or 17 or 18 or 19 or 20 or 21 or 22 or 23 or 24 or 25 or 26
28. 4 and 27
29. Limit 28 to English
30. Publication years 2005–current

We divided the search terms into 2 themes (“intravenous medication therapy” and “medication errors”), both of which needed to appear in the included articles. The theme medication error was chosen according to our study objectives to explore preventable adverse drug events, which occur as a consequence of errors in the medication process caused by omissions or commissions.^6,29^ The search strategy was completed with other terms similar to medication error (Table 1), as inconsistency in terminology and definitions related to medication errors is widely known.^30^ A combination of the themes “adverse drug event” and “intravenous” was also considered. It was not included in the final search strategy because the combination resulted in a significantly large number of citations with an emphasis on drug safety and adverse drug reactions without objectives relating to medication safety and the medication use process. We supplemented the search with a manual search of the reference lists of the included articles to identify all relevant publications.

### Inclusion and Exclusion Criteria

We applied a predetermined PICO tool (participants, interventions, comparison, and outcomes) to select studies for inclusion.^25^ A study was included if participants were hospitalized patients or the study used a patient scenario in a simulated hospital environment and patients received IV medication. We decided to include simulation studies because clinical simulation enables the assessment of new systemic defenses in a safe and controlled environment without risk of patient harm.^31^ We excluded studies conducted in ambulatory settings, such as home infusion chemotherapy, as we wanted to focus on in-hospital IV medication processes. We also excluded studies focusing on multiple administration routes, if the findings related to IV administration could not be reliably identified and extracted from the results. Comparison was not required, which means that we included studies using both controlled and uncontrolled study designs. Studies applying measures associated with the assessment of systemic defenses intended to prevent IV medication errors and/or systemic causes resulting in medication errors were included. Studies exploring unpreventable adverse drug events or only incidence and types of medication errors were excluded. Only English language articles were included. Peer-reviewed journal articles using all methods and study designs were included.

### Study Selection

**FIGURE 1:**
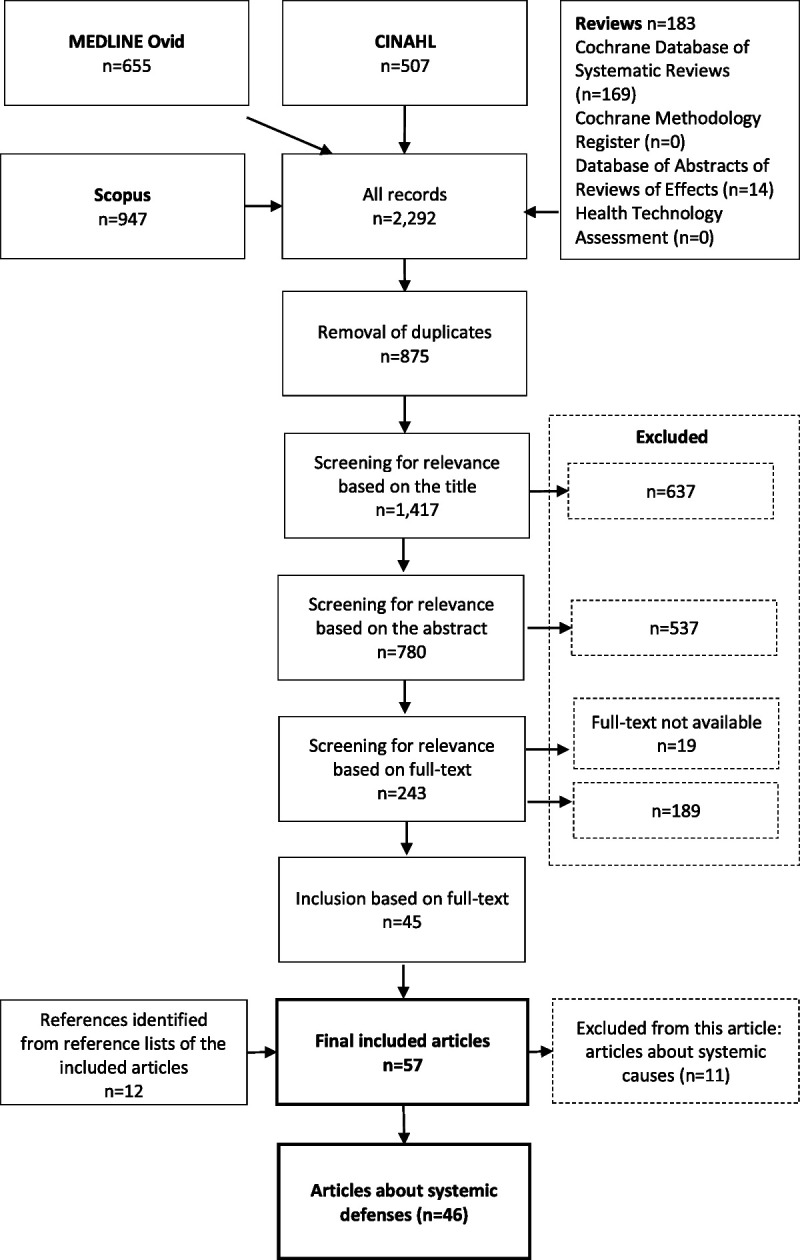
Flowchart of the study.

After the removal of duplicates, the search produced 1417 potentially relevant publications (Fig. 1). Two reviewers (S.K.K., I.N.) independently selected studies based on the titles. In case of disagreement, the article was included in the next phase, in which the reviewers (S.K.K., I.N.) independently selected studies based on the abstracts. Disagreements were resolved through discussion and consensus with a third reviewer (A.-R.H.). The reviewers (S.K.K., I.N.) independently selected studies based on full texts of the remaining publications. The articles fulfilling the inclusion criteria of both reviewers were included (n = 36). Disagreements were resolved through discussion and consensus with the third reviewer (A.-R.H.), which led to the inclusion of 9 more articles. A total of 45 publications met the inclusion criteria. After this, reference lists of the included articles were searched manually for relevant articles (n = 12), giving us a total of 57 included studies.

We identified 2 major themes among the selected articles: systemic defenses aiming to prevent errors and systemic causes of in-hospital IV medication errors (Fig. 1). The articles focusing on systemic defenses for preventing IV medication errors (n = 46) are reported in this publication. Articles focusing on systemic causes of IV medication errors are discussed in another publication.

### Data Extraction and Analysis

Data extraction and analysis were carried out by 2 of the authors (S.K.K., I.N.), and the results were carefully reviewed by the other authors (A.-R.H., M.A.). Study characteristics, country, study design, setting, evidence quality, systemic defense and comparison, number of patients (or other), primary measures, and key findings were extracted to a table (Supplementary File 1, http://links.lww.com/JPS/A280). We assessed the quality of the evidence using the GRADE system, which has the following 4 levels of evidence quality: very low, low, moderate, and high.^26^ Evidence from randomized controlled trials (RCTs) and systematic reviews was graded as high quality, and evidence that included observational data was graded as low quality. For example, observational studies conducted in a simulated environment with a small sample size were graded as low quality. Factors that decreased the quality of evidence (e.g., study limitations and inconsistency of results) or increased the quality of evidence (e.g., large magnitude of effect such as a large sample size, controlled study design, and multiple data collection methods and sources such as smart pump–produced log reports, chart reviews, staff reports, and incident reports) were also taken into account. Primary measures used in the articles concerning systemic defenses for preventing IV medication errors were extracted to Table 2 to demonstrate methodological variation between the included studies.

**TABLE 2:** Synthesis of the Primary Measures Used in the Included Studies (n = 46)

Measures used in more than one study
Medication errors (n = 25)
Detection methods: direct observation (n = 8),^32–39^ self-reporting (n = 4),^40–43^ patient record review (n = 4),^44–47^ analyses of infusion concentrations (n = 3),^48–50^ medication record review (n = 1),^51^ order review (n = 1),^52^ observation of infusion labels (n = 1),^53^ automated compounding workflow system (n = 1),^54^ self-reporting in the control group and errors detected by the system in the intervention group (n = 1),^55^ self-reporting in the control group and drug chart review in the intervention group (n = 1)^56^
Time to task completion (n = 12)
Detection methods: direct observation (n = 9),^34,36–39,49,53,56,57^ electronic system time-stamps (n = 2),^47,55^ self-reporting (n = 1)^58^
Adverse drug events^59^ and clinical incidents^43,58,60,61^ (n = 5)
Detection methods: self-reporting (n = 2),^43,61^ patient monitoring (n = 2),^58,60^ self-reporting and automatic surveillance (n = 1)^59^
Potentially prevented medication errors (n = 4)
Detection methods: smart pump alert log data (n = 3),^62–64^ computerized physician order entry (CPOE) alert log data (n = 1)^65^
Medication errors recognized by the participants in simulated scenarios (n = 4)
Detection methods: direct observation (n = 4)^34,36,57,66^
Serious medication errors (n = 2)
Detection methods: direct observation (n = 1),^39^ multiple methods (n = 1)^67^
Compliance in drug library use (n = 2)
Detection methods: smart pumps alert log data (n = 2)^63,64^
Incompatible drug pairs (n = 2)
Detection methods: drug chart review (n = 2)^68,69^
Measures used in only one study
Measures related to medication errors: incidence of inappropriate prescribing,^70^ medication error type,^52^ order completeness,^44^ incompatible pantoprazole combinations,^69^ accuracy of drug identification and concentration verification^71^
Measures related to feasibility of a systemic defense: benefits of a systemic defense,^24^ negative effects of a systemic defense,^24^ feasibility of a systemic defense,^72^ consensus equal to or more than 80%^73^
Measures related to medication therapy and medication use process: incidence of good glucose control,^74^ time between first glucose control to insulin initiation,^74^ 24-h cumulative haloperidol dose,^75^ incidence of diagnostic tests,^75^ acyclovir dilution volume (in milliliters),^76^ number of ampoules and premixed infusions dispensed to the ward,^61^ potentially risky practices^67^

We analyzed the included articles using qualitative content analysis to identify systemic defenses and their ability to prevent IV medication errors.^27,28^ The findings were extracted and classified according to which medication process stage was most affected by the systemic defense mechanism (Tables 3, 4). The systemic defenses, evidence quality, and key findings are presented in Table 3. We assessed the statistical significance of the key findings according to possible statistical analysis presented in the articles, such as *P* value (*P* < 0.05) and confidence interval (95% confidence interval excludes the null value). Key conclusions and recommendations presented by the authors were extracted to Table 4.

**TABLE 3:** Systemic Defenses, Evidence Quality, Key Findings, and Statistical Significance of the Findings in the Included Studies (n = 46)

Systemic Defense and Evidence Quality	Key Findings (Statistically Significant/*Not Significant or Significance Not Reported*)
Prescribing (n = 8)	
CPOE and CDSS (n = 2)	
Targeted alert for IV haloperidol (versus no alert) L^70^	Decreased inappropriate prescribing (50% versus 14%; average of 4.1/mo to 1.5/mo)^70^
Pediatric resuscitation orders (versus handwritten orders) L^56^	Reduced time to order completion (14 min 42 s versus 2 min 14 s) and *elimination of errors (3 versus 0)*^56^
Online dosing calculators and CDSS (n = 2)	
Complex dosing for obese patients (versus manual) L^46^	Decreased frequency calculation errors (12.8% versus 4%) and *prescribing errors (43% versus 20%)*^46^
Pediatric continuous infusions (versus manual) L^52^	83% fewer orders containing ≥1 errors (55% versus 6%) and elimination of high-risk errors (26% versus 0%)^52^
Standard order form (n = 2)	
Pediatric resuscitation room (versus before) M^44^	Increased order completeness (5% versus 33%) and decreased prescribing errors (15% versus 6%)^44^
KCl infusions (versus before) M^60^	Decreased postinfusion serum potassium elevations (7.7% versus 0%) and infusions administered to patients with high serum potassium (2.9% versus 0.0%)^60^
Order verification by pharmacist present (n = 1; versus in hospital pharmacy) L^47^	*Patients received appropriate first antibiotic 93.4% of the time (versus 86.3%)* and second 96.8% of the time (versus 83.3%). Time from order to verification for the first 2 doses was shorter (10.5 min versus 11.4 min).^47^
Multidisciplinary intervention to improve IV PPI prescribing* (n = 1; versus before) L^45^	In 2 patient groups, 26% and 41% reduction in patients without an appropriate indication^45^
Dispensing (n = 1)	
CPOE infusion orders with standard concentrations (versus handwritten orders versus handwritten orders with errors; n = 1) L^53^	Infusions processed from CPOE orders contained fewer errors (4% versus 26% versus 45%). Processing CPOE orders required less time.^53^
Preparation (n = 6)	
Compounding workflow software (n = 2)	
Automated workflow management system (no comparison) L^54^	*Total error rate of 0.74%, of which the system detected 72.27% of errors (incorrect drug/diluent), and pharmacist’s inspection of 27.73% (wrong volume/damaged product)* ^54^
Gravimetric workflow software system (versus manual compounding) L^55^	*Higher error rate detected by the system (7% versus 0.096%). Barcode scanning detected 26% of the total errors; the gravimetric weighing, 71%; and vial reconstitution, 3%.* ^55^
Automated infusion production in pharmacy (n = 1; versus ward-based preparation) L^48^	The mean concentration was closer to the target in machine-made solutions (101.1% versus 97.2%). *Decrease in ≥5% (53% versus 16%) and >10% (22% versus 5%) deviations*^48^
Prefilled syringes for emergency situation (n = 1; versus preparing drug infusions at the bedside) L^49^	Decreased time for the infusion to be started (276 s versus 156 s, a mean delay of 106 s). Errors were 17.0 times less likely with prefilled syringes. Infusions prepared by pharmacy and industry were more likely to contain the right concentration.^49^
Standard concentrations, preparation protocols, and education (n = 1; versus before) L^50^	Accuracy error rate decreased both in NICUs (54.7% versus 23%) and hospital pharmacy (38.3% versus 14.6%). *Calculation errors decreased in NICUs (1.35% versus 0%). No calculation errors in hospital pharmacy samples*^50^
Automated quality check with tabletop-enhanced photoemission spectroscopy for IV admixtures (n = 1; no control) L^71^	*The device detected errors departing from the targeted concentration ≥20% with a sensitivity of ≥95%. Specificity in distinguishing among test medications at targeted concentrations was 100%.* ^71^
Administration (n = 24)	
Smart infusion pumps (n = 11)	
Systematic review of benefits and risks of smart pumps (n = 1) H^24^	Smart pumps with only soft limits^51,66,67^ and both hard and soft limits^42,58^ are unlikely to reduce IV medication errors. *In uncontrolled studies, 4.8%–14% of soft alerts led to averted errors.*^62,63,65^
Smart pumps with drug library (versus drug library off; n = 1) M^67^	Decrease in wrong patient errors with smart barcode pump (88% versus 58% versus 46%) and wrong dose hard limit errors with smart pump and smart barcode pump (79% versus 75% versus 38%).^66^
Smart pumps with drug library (versus conventional pumps; n = 3) L^40,42,51^	Implementation of standard concentrations, smart pumps, and new labels resulted in a 73% reduction in error rate (0.8 versus 3.1/1000 doses, an absolute risk reduction of 2.3/1000 doses).^40^
Smart pump with barcode (versus smart pump versus conventional infusion pump; n = 1) L^66^	Decreased incidents related to changeover of vasoactive infusions (20% versus 11%).^58^
Automated changeover of vasoactive drug infusion pumps (versus manual changeover; n = 1) L^58^	*According to a systematic review, the benefits of smart pumps are intercepting errors (e.g., wrong rate, dose, or pump settings), reduction of adverse drug events, practice improvements, and cost-effectiveness. Issues related to smart pumps were lower compliance rates, the overriding of soft alerts, nonintercepted errors, and the possibility of using the wrong drug library.* ^24^
Smart pumps with drug library (n = 3) L^62–64^ or electronic medical record smart system to notify of pump programming errors (n = 1) L^65^ (no comparison)	*The compliance in drug library use reported in the studies has been variable and insufficient (62%–98%).* ^24,63,64,67^
Color-coded safety systems (n = 3)	
Color-coded prefilled syringes for pediatric resuscitations (versus before) L^39^	Decreased time to medication administration (47 s versus 19 s) and decrease in critical dosing errors (17% versus 0%).^39^
Pediatric emergency system* (versus before) L^36^	Error reduction in dose conversion (25.6% versus 2.5%), dilution (35.6% versus 0.63%) and administration (54.7% versus 3.9%). Reduced median time to task completion (109 s versus 28 s).^36^
Color-coded labels for emergency infusion fluids (versus before) L^37^	Time improvement in all scenarios. *Decreased wrong fluid errors (13 versus 0).*^37^
Anesthesia safety system (n = 2)^†^ (versus before intervention) H,^35^ M^43^	Decreased overall error rate (11.6 versus 9.1 errors/100 administrations). Lower error rate when barcode scanning before administration and keeping the voice prompt active were applied than when not applied (6.0 versus 9.7 errors/100 administrations).^35^
	Decreased errors (0.049% versus 0.032%; a relative reduction of 35%) and major adverse outcomes from errors (0.002% versus 0%).^43^
Standard operating procedure to prevent IV incompatibilities (n = 2; versus before) L^68,69^	Reduction of incompatible drug pairs (5.8% versus 2.4%) and incompatible drug pairs that were governed by the new procedure (1.9% versus 0.5%).^68^
Administration guidelines (n = 2)	Decrease in incompatible pantoprazole combinations (100.0% versus 56.2%).^69^
Checklist to detect errors (versus old checklist) L^33^	Increased overall error detection (38% versus 55%) and detection of identification errors (80% versus 15%). No significant difference in error detection related to pump programming, mismatch or clinical decisions.^33^
Algorithms for pediatric chemotherapy (no control) L^73^	*The agreement in Delphi validation was 92.8%–99.0%. The algorithms are valid to prevent and manage antineoplastic agents’ extravasation.* ^73^
CPOE-generated infusion orders with standard concentrations (n = 1; versus handwritten orders) L^57^	Nurses were able to check the accuracy of pump settings in less time (6 min 18 s ± 2 min 26 s versus 8 min 47 s ± 3 min 6 s), but CPOE did not improve the ability to detect pump programming errors.^57^
Barcode drug verification (n = 1) (versus 2-person confirmation) L^72^	*Both methods were perceived to contribute to the prevention of errors, but barcode scanning is more feasible. There are limitations related to 2-person confirmation (e.g., continuous presence of the second person, no distraction, or time pressure).* ^72^
Calculator to convert orders to volumes and administration rates (n = 1; versus no intervention) L^34^	Increased medication volumes calculated and drawn accurately (91% versus 61%) and correct recall of essential medication information (97% versus 45%), better recognition of unsafe doses (93% versus 19%). Reduced calculation times (1.5 min versus 1.9 min)^34^
Interventions to prevent errors caused by interruptions^‡^ (n = 1; versus no interventions) L^32^	Decreased error rate when interrupted during verification of syringe drug volumes (89% versus 58%), verification of drug volumes programmed in ambulatory pumps (94% versus 58%), IV push (89% versus 32%), and pump programming (39% versus 5%)^32^
Treatment monitoring (n = 2)	
CPOE and CDSS (n = 2)	
IV insulin protocol (versus manual protocol) L^74^ CPOE set for IV haloperidol treatment monitoring (versus before) L^75^	Reduced time from first glucose measurement to insulin initiation (2–3 d versus 12 h). Improved amount of all glucose readings in ideal range (29.3% versus 37.7%) and time spent in ideal range by patients on IV insulin for >24 h (116 min/d)^74^
	Patients were more likely to have 24-h cumulative dose <2 mg (47.8% versus 64.3%), baseline ECG (65.5% versus 80.6%), follow-up ECG within 24 h of administration (25.2% versus 58.5%), and Mg value assessed at time of administration (51.2% versus 74.6%).^75^
Standardization of high-risk medication use process (n = 5)	
Interdisciplinary intervention to increase dilution of IV acyclovir (versus before) L^76^	The median volume in which the acyclovir dose was administered was significantly higher in the postintervention group (250 mL versus 100 mL).^76^
Safety intervention in IV potassium use (versus before) L^61^	The number of incidents was significantly reduced from 23 to 9, and the *number of ampoules dispensed was reduced from 10, 100 to 0.*^61^
Computerized continuous IV insulin protocols for tight glycemic control (versus paper protocol) L^38^	Fewer errors in the titration (13 versus 113) and transition phases (9 versus 23), *fewer dosing errors in the initiation phase,* and less time to complete the titration (6 versus 9.5 min)^38^
PCA safety intervention (versus before) M^41^	The odds ratio of a PCA error after intervention was 0.28 (95% CI, 0.14–0.53) and the odds ratio of a pump-programming error was 0.05 (95% CI, 0.001–0.30).^41^
CDSS, CPOE, and PCA smart pumps (versus before) M^59^	*Decrease in PCA events detected by automated surveillance (22%; 4.2 versus 5.3/1000 PCA days)* and voluntary report system (72%; 0.66 versus 2.4/1000 PCA days)^59^

Italics to indicate if the results were not statistically significant or significance was not reported.

Evidence quality: L, low; M, moderate; H, high.

*Color-coded weight zones, precalculated doses, and directions for administration, preparation, and monitoring.^36^

^†^Drug trays and trolley, prefilled syringes, color-coded labels, barcode drug verification and administration record, and safety alarms.^35,43^

^‡^Verification: verification booth, standard workflow, and speaking aloud; administration: visual timers for IV pushes, no interruption zones, speaking aloud, and reminder signage.^32^

CDSS, clinical decision support system; ECG, electrocardiogram; IV, intravenous; NICU, neonatal intensive care unit; PCA, patient-controlled analgesia; PPI, proton pump inhibitors.

**TABLE 4:** Conclusions and Recommendations Presented by the Authors of the Included Studies (n = 46)

Process Stage	Key Conclusions and Recommendations
Prescribing (n = 8)	A standard order form increases order completeness and reduces prescribing errors and patient harm.^44,60^
	Online calculators improve prescribing in complex dosing policies (e.g., obese and pediatric patients)^46,52^ and eliminate high-risk errors.^52^
	A customized alert significantly decreased inappropriate prescribing, but providers may abandon an appropriate prescription in response to an alert.^70^
	CPOE- and CDSS-generated resuscitation orders are legible, complete, automatically checked for accuracy, and completed in less time.^56^
	When a pharmacist is present, patients are more likely to receive appropriate doses of antimicrobials and in a more timely fashion.^47^
	A multidisciplinary approach involving simple interventions resulted in improved physician prescribing behavior.^45^
Dispensing (n = 1)	CPOE orders saved pharmacists’ time and improved the safety of processing continuous infusions, although not all errors were eliminated.^53^
Preparation (n = 6)	Compounding workflow software systems (e.g., barcode scanning, gravimetric weighing of components, and real-time images of process steps) improve detection of preparation errors.^54,55^
	Centralized, automated preparation of standardized infusion solutions may be an effective means for reducing clinically relevant deviations in concentration conformity of infusion solutions.^48^
	Providing drug infusions in syringes prefilled by pharmacists or pharmaceutical companies would reduce medication errors and treatment delays.^49^
	Calculation errors can disappear with good standardization protocols, but a decrease in accuracy error depends on good preparation techniques and environmental factors.^50^
	A tabletop EPS device demonstrated sensitivity and specificity in validating the identity and concentrations of high-risk IV medications and may help prevent medication errors caused by inaccurate compounding.^71^
Administration (n = 24)	Smart pumps reduce but do not completely prevent pump programming errors.^24,40,58,62–66^ High override rates of soft limits and insufficient compliance in drug library use limit the effectiveness.^24,42,51,63,64,66,67^ Hard limits play a main role in intercepting errors.^24,66^ Opportunities for improvement include integrating smart pumps with barcode readers and CPOE real-time clinical data (e.g., glucose control and respiratory monitoring).^24,51,62–66^ Smart pumps allowing automated relays of vasoactive infusion pumps reduce hemodynamic incidents.^58^
	Color-coded systems such as prefilled syringes,^39^ pediatric weight zones,^36^ and labels^37^ decrease time to medication administration^36,37,39^ and reduce pediatric errors^36,39^ and wrong fluid errors^37^ in simulated emergency situations.
	Anesthesia safety systems including drug trays and trolley, prefilled syringes, color-coded labels, barcode drug verification, and administration record and safety alarms reduce medication errors^35,43^ and adverse outcomes.^43^
	Administration of incompatible drugs in intensive care can be reduced by procedural interventions with standard operating procedure.^68,69^
	Checklists designed with explicit step-by-step instructions are useful for detecting errors when a care provider is required to perform a long series of mechanistic tasks under a high cognitive load.^33^
	Standardization of high-risk medication use (e.g., validated algorithms for extravasation prevention in pediatric peripheral chemotherapy) can enhance patient safety by establishing rapid intervention and proper follow-up.^73^
	The use of CPOE-generated orders for continuous infusions saved nurses’ time and improved user satisfaction but did not decrease the incidence of medication errors associated with verification of infusion pump settings.^57^
	Barcode scanning is more feasible than 2-person confirmation when verifying use of the right drug.^72^
	A calculator to convert orders to volumes and administration rates ﻿improved nurses’ performance in drug calculations during simulated clinical scenarios.^34^ Interventions can reduce unanticipated errors of commission in medication administration tasks when interruptions occur, but effectiveness at reducing predictable errors of detection in medication verification tasks is mixed.^32^
Treatment monitoring (n = 2)	Integrating a computer-based insulin protocol into a CPOE system achieved efficient, safe, and effective glycemia control in surgical intensive care unit patients.^74^
	The use of a CPOE set improved treatment monitoring when prescribing IV haloperidol (e.g., electrocardiogram and electrolyte monitoring) and reduced the proportion of subjects who received haloperidol >2 mg/24 h.^75^
Standardization of a high-risk medication use process (n = 5)	Technology (CPOE, CDSS, PCA smart pumps)^59^ and safety interventions (e.g., standardized orders, education, and independent manual double checks)^41^ decrease PCA-related medication errors.
	Use of an easily applied intervention increased the amount of IV fluid administered to patients receiving acyclovir, a potentially nephrotoxic medication.^76^
	In a simulated environment, a computerized protocol for tight glycemic control resulted in significant insulin dosing error reduction, saved time and improved nurse satisfaction.^38^
	A multifactorial approach to the safe prescribing, dispensing, and administration of IV potassium reduced the potential for patient harm.^61^

CDSS, clinical decision support system; EPS, enhanced photoemission spectroscopy; PCA, patient-controlled analgesia.

## RESULTS

### Characteristics and Main Outcomes of Included Studies (n = 46)

Our systematic review is based on 46 peer-reviewed original articles (Supplementary File 1, http://links.lww.com/JPS/A280).^24,32–76^ The studies were conducted in 11 countries, which included the United States (n = 22),^24,34,36,38–40,47,51–55,57,59,60,62,65,67,70,71,74,75^ Canada (n = 8),^32,33,41,42,44,45,66,76^ Germany (n = 4),^48,63,68,69^ the United Kingdom (n = 3),^46,49,72^ New Zealand (n = 2),^35,43^ Spain (n = 2),^50,64^ Australia (n = 1),^61^ Brazil and the United States (n = 1),^73^ France (n = 1),^58^ Israel (n = 1),^56^ and Korea (n = 1).^37^ Altogether, 30 studies (65%) were carried out in North America. Most of the studies were conducted in a hospital setting (n = 34),^35,40–48,50–52,54–56,58–65,67–76^ and some in simulated hospital environments (n = 11).^32–34,36–39,49,53,57,66^ One study was a systematic review including studies conducted both in a hospital setting and in a simulated hospital environment.^24^

There was a lot of variations between the study designs and the evidence quality of the studies. Of the 46 included articles, 38 (83%)^32–53,55–61,66–70,72,74–76^ involved a controlled study design. Only 2 studies (4%) were graded as high quality: of these 2, one applied an RCT design^35^ and the other was a systematic review.^24^ Six studies (13%)^41,43,44,59,60,67^ used a controlled observational study design with large magnitude of effect, which is why they were graded as moderate quality. Four of these were analyses of incident reports, medication error reports, or adverse drug event data,^41,43,59,67^ and 2 were observational reviews of patient records.^44,60^ The remaining 38 studies (83%)^32–34,36–40,42,45–58,61–66,68–76^ applied an observational study design without large magnitude of effect, which is why they were graded as low quality. Controlled low-quality studies (n = 31) applied variable designs: simulation studies (n = 11)^32–34,36–39,49,53,57,66^; observational reviews of drug charts, medication orders, or patient records (n = 11)^45–47,51,52,68–70,74–76^; studies combining multiple methods (n = 4)^56,58,61,72^; analyses of medication error or adverse drug event data (n = 3)^40,42,55^; and analyses of infusion concentrations (n = 2).^48,50^ Some low-quality studies (n = 7)^54,62–65,71,73^ used an uncontrolled study design. The study limitations were not reported, and their influence was not assessed in 5 studies (11%).^41,46,58,62,73^

The primary measures used in the included studies varied, but we identified some shared measures (Table 2). The measure most widely used to assess the effectiveness of a systemic defense was incidence of medication errors, which appeared in 25 studies (54%).^32–56^ There was a lot of variations between the error detection methods used, which makes it difficult to compare results between the studies. Measures quite similar to medication errors, such as adverse drug events and clinical incidents (n = 5),^43,58–61^ potentially prevented medication errors (n = 4),^62–65^ and serious medication errors (n = 2)^39,67^ were used in 11 studies (24%). Time to task completion appeared in 12 studies,^34,36–39,47,49,53,55–58^ and it was a commonly used measure especially in simulation studies (n = 9).^34,36–39,49,53,57^

### Systemic Defenses and Their Ability to Prevent Intravenous Medication Errors

Systemic defenses, their ability to prevent IV medication errors, and statistical significance of the key findings are presented in Table 3. Key conclusions of the included studies and recommendations presented by the authors are presented in Table 4. Of the systemic defenses identified, most were related to administration (n = 24 studies; 52%),^24,32–37,39,40,42,43,51,53,57,58,62–69,72,73^ followed by prescribing (n = 8; 18%),^44–47,52,56,60,70^ preparation (n = 6; 13%),^48–50,54,55,71^ treatment monitoring (n = 2; 4%),^74,75^ and dispensing (n = 1; 2%).^53^ Five studies^38,41,59,61,76^ (11%) focused on high-risk process standardization and involved implementation of systemic defenses related to multiple drug delivery process stages.

**FIGURE 2:**
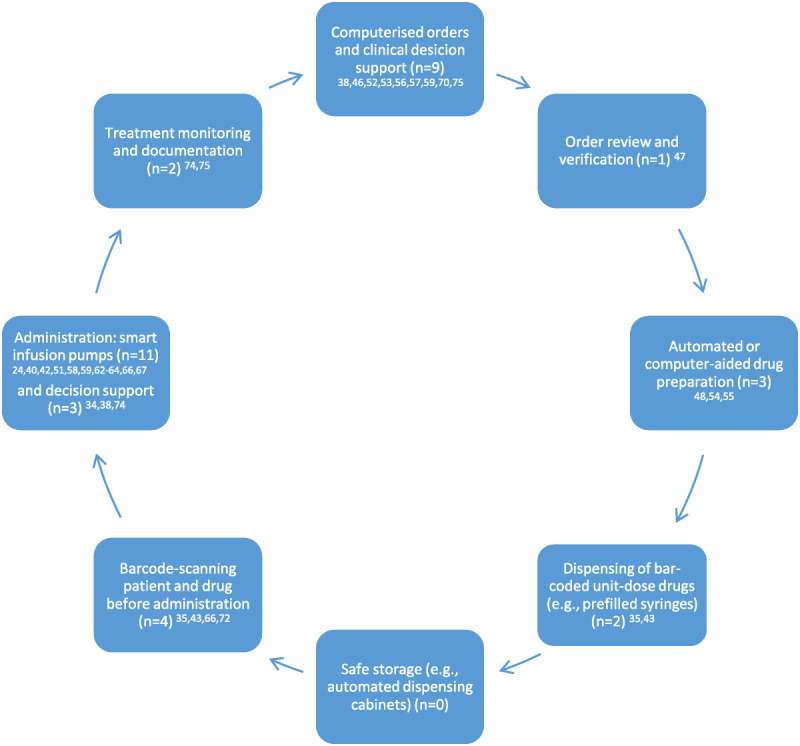
Systemic defenses related to closed-loop medication management explored in the included studies (n = 46; modified from Refs.^13–16,20^).

Systemic defenses, including features of closed-loop medication management systems, appeared in 61% of the studies (n = 28; Fig. 2),^24,34,35,38,40,42,43,46,48,51–59,62–67,70,72,74,75^ with smart pumps being the systemic defense most widely studied (n = 11; 24%).^24,40,42,51,58,62–67^ Besides preventing prescribing errors, computerized orders and decision support systems were found to contribute toward safe dispensing,^53^ administration,^34,38,57^ and treatment monitoring^38,74,75^ by preventing errors related to interpretation of orders, calculation tasks, and follow-up. In addition to systemic defenses related to closed-loop medication management systems, prefilled syringes^39,49^ and color-coded systems^36,37,39^ were found to reduce errors in high-risk environments and situations, such as operating rooms and resuscitation.

Although smart infusion pumps were the systemic defense most widely studied, their effectiveness in medication error prevention remains unclear (Tables 3, 4). The key component of smart pump is a drug library containing predefined parameters for the drug type, strength, and dosing limits of specific drugs. Soft limits are alerts that can be overridden by clinicians, whereas hard limits cannot be overridden. Insufficient compliance in drug library use is problematic, as the systemic defense is not active if drug library is bypassed.^24,63,64,67^ Another issue is high override rate of soft limits, which, unlike hard limits, do not require changes to pump programming when the patient is at risk of getting a wrong dose.^42,51,58,62,63,65–67^ Opportunities for improvement include use of hard limits and integrating smart pumps with other systemic defenses, such as barcode readers and computerized physician order entry (CPOE) real-time clinical data (e.g., glucose control and respiratory monitoring).^24,51,62–66^

## DISCUSSION

To the best of our knowledge, this is the first systematic review to summarize systemic defenses and their ability to prevent IV medication errors in hospitals. We found 46 studies involving variable systemic defenses, study designs, and evidence quality. There were 2 high-quality studies and 6 observational studies with large magnitude of effect. Within the included articles, most studies applied an observational study design without large magnitude of effect and did not provide the most rigorous evidence. More than 50% of the studies focused on administration stage, with smart infusion pumps being the most widely studied systemic defense (n = 11). We found a limited number of studies exploring other stages of medication use process; all of them were observational low- or moderate-quality studies that did not provide the most rigorous evidence. Systemic defenses involving features related to closed-loop medication management systems were explored in 28 of 46 studies.

According to our findings, smart infusion systems reduce, but do not completely prevent, pump programming errors,^24,40,58,62–66^ which has also been stated in an earlier systematic review.^24^ We identified high override rates of soft limits and insufficient compliance in drug library use as key limitations for effectiveness.^24,42,51,63,64,66,67^ To make smart pumps more effective and thus prevent pump programming errors, increasing the use of hard limits in the drug libraries is important.^24,66^ Another area of development is the functionality of smart pumps and drug libraries, as a recent study found differences in smart pump compliance both within and between hospital systems, which might be influenced by pump type and the number of drug library profiles.^77^ Prevention of errors throughout the IV medication process requires integrating smart pumps into closed-loop medication management systems, such as electronic patient records, clinical pharmacist’s review of orders, automated compounding systems, barcode verification at the bedside, and real-time clinical monitoring data.^20,24,48,49,51,54,55,62–66,78^

A significant error reduction was reached in one of the high-quality studies, which was an RCT study exploring a system designed to reduce errors in the recording and administration of drugs in anesthesia.^35^ The same system was also studied in another study included in our systematic review, and it involved drug trays and a drug trolley, prefilled syringes, color-coded labels, barcode drug verification and administration records, and safety alarms to support safe drug administration.^35,43^ Color-coded systems^36,37,39^ and prefilled syringes^39,49^ also showed effectiveness in other studies by reducing errors and time to medication administration in simulated emergency situations. In the future, it is important to ensure the availability of barcoded unit-dose medications to simplify the IV drug delivery process in the clinical area. In many countries, bar-coded unit-dose medications are not yet commercially available and most of the IV drug preparation is carried out by nurses and pharmacists in the ward environment, where errors are more likely to happen.^2,79,80^

Five of the included studies^38,41,59,61,76^ focused on high-risk medication process standardization and involved systemic defenses in multiple stages of the drug delivery process, which is what the Institute for Safe Medication Practices recommends to support resolving medication safety issues related to high-alert medications.^3^ Another reason to study larger parts of the drug delivery process is to find out how different systemic defenses work together and, on the other hand, how one systemic defense can affect multiple process stages. As an example, in addition to preventing prescribing errors, computerized orders and decision support systems were found to contribute to safe dispensing, administration, and treatment monitoring by preventing errors related to interpretation of orders, calculation tasks, and treatment monitoring.^34,38,53,57,74,75^

Our study was conducted in accordance with the Preferred Reporting Items for Systematic Reviews and Meta-Analyses checklist.^25^ We included only peer-reviewed articles in the analysis, and the quality of selected studies was assessed using the GRADE system.^26^ In addition, we extracted and evaluated the statistical significance of the results presented in the included studies. The literature search was restricted to articles published in English; thus, studies published in other languages were excluded. However, our study indicated that most of current evidence in this research area has been published in English-speaking countries, especially in North America.

This systematic review has several limitations. The quality of included studies was relatively low, as most studies (44/46) applied observational methodologies. The studies used different measures and study designs, which is why quantitative analysis was not performed. Incidence of medication errors was a commonly used measure, but there was variation between the error detection methods. None of the studies used more than 1 error detection method, which has been recommended for discovering representative information concerning medication errors.^81^ Because the data were not summarized statistically, we decided to include an earlier systematic review by Ohashi et al^24^ to the analysis. If quantitative analysis could have been performed, double counting the articles included both in our study and in the systematic review by Ohashi et al^24^ (n = 9)^40,41,51,59,63–67^ would have been a more critical source of bias. Most included studies focused on the administration stage, probably due to administration being the most error-prone stage of the IV medication process.^6,18,19^ The number of studies covering other medication use process stages was limited, which might be due to the fact that the studies exploring other phases might involve multiple administration routes. As an example, none of the included studies explored automated drug distribution systems, which have been indicated to improve medication safety.^16^

We had to exclude some promising articles because they seemed to be descriptive project reports and lacked a scientific study design, which might indicate that this research area is still under development. This is why our decision to study systemic defenses in all hospital environments was a good choice, as many defenses can be modified and applied in different care settings. An interesting area for further studies is to explore systemic defenses related to IV medication in certain care environments, medical specialties, and patient groups. Eleven studies were conducted in a simulated environment, and it is important to examine these defenses in real life as well. Future studies should explore combinations of systemic defenses and their effectiveness in error prevention in multiple stages of the drug delivery process. As new technology is implemented and more data are available from the systems, it is essential to use this information to assess the effectiveness and areas of development. There is also a need to explore systemic defenses in other settings than inpatient care, whereas IV administration is increasingly common in ambulatory settings.

## CONCLUSIONS

Most included studies focused on the administration stage, with smart infusion pumps being the most widely studied systemic defense. We also found a limited number of studies exploring other stages of the medication use process. Most of the systemic defenses involved features related to closed-loop medication management systems. Our study provides health care organizations with preliminary knowledge about systemic defenses intended to prevent IV medication errors, but more rigorous evidence is needed. There is a need for further studies to explore combinations of systemic defenses and their effectiveness in error prevention.

## Supplementary Material

SUPPLEMENTARY MATERIAL
