# Supplementary material for: Systemic Defenses to Prevent Intravenous Medication Errors in Hospitals: A Systematic Review
Source: J Patient Saf. 2020 Mar 17;17(8):e1669–80. doi: 10.1097/PTS.0000000000000688 (PMC8612901; doi:10.1097/PTS.0000000000000688)
Supplement: SUPPLEMENTARY MATERIAL [file pts-17-e1669-s002.docx]

**SYSTEMIC DEFENSES TO PREVENT INTRAVENOUS MEDICATION ERRORS IN HOSPITALS: A SYSTEMATIC REVIEW**

Sini Kuitunen (corresponding author), MSc (Pharm), ﻿HUS Pharmacy, Hospital Pharmacy of Helsinki University Hospital (HUS) and PhD student, Clinical Pharmacy Group, Faculty of Pharmacy, University of Helsinki, Finland

Ilona Niittynen, MSc (Pharm), Clinical Pharmacy Group, Faculty of Pharmacy, University of Helsinki, Finland

Marja Airaksinen, PhD (Pharm), Clinical Pharmacy Group, Faculty of Pharmacy, University of Helsinki, Finland

Anna-Riia Holmström, PhD (Pharm), Hospital Pharmacy of Helsinki University Hospital (HUS) and Clinical Pharmacy Group, Faculty of Pharmacy, University of Helsinki, Finland

**Correspondence:** Sini Kuitunen, MSc (Pharm), ﻿HUS Pharmacy, Hospital Pharmacy of Helsinki University Hospital (HUS); PhD student, Clinical Pharmacy Group, Faculty of Pharmacy, University of Helsinki, Finland. (e-mail: sini.kuitunen@hus.fi, tel. +358407704394)

**Conflicts of Interest and Source of Funding:** Sini Kuitunen received a PhD research grant from the University Pharmacy (Yliopiston Apteekki), which is a pharmacy corporation owned by the University of Helsinki, Finland. The authors disclose no conflicts of interest.

**ABSTRACT**

**Objectives:** Intravenous medication delivery is a complex process that poses systemic risks of errors. The objective of our study was to identify systemic defenses that can prevent in-hospital intravenous medication errors.

**Methods:** A systematic review adhering to PRISMA guidelines was conducted. We searched MEDLINE (Ovid), Scopus, Cinahl and EMB reviews for articles published between January 2005 and June 2016. Peer-reviewed journal articles published in English were included. Two reviewers independently selected articles according to a predetermined PICO-tool. The quality of studies was assessed using the GRADE system and the evidence analyzed using qualitative content analysis.

**Results:** 46 studies from 11 countries were included in the analysis. We identified systemic defenses related to administration (n=24 studies), prescribing (n=8), preparation (n=6), treatment monitoring (n=2), and dispensing (n=1). In addition, five studies explored defenses related to multiple stages of the drug delivery process. Systemic defenses including features of closed loop medication management systems appeared in 61% of the studies, smart pumps being the defense most widely studied (24%). The evidence quality of the included articles was limited, as 83% were graded as low quality, 13% moderate quality, and only 4% high quality.

**Conclusions:** In-hospital intravenous medication processes are developing toward closed loop medication management systems. Our study provides healthcare organizations with preliminary knowledge about systemic defenses that can prevent intravenous medication errors, but more rigorous evidence is needed. There is a need for further studies to explore combinations of different systemic defenses and their effectiveness in error prevention throughout the drug delivery process.

**Keywords**: patient safety; medication safety; intravenous medication; medication error; systemic defense; risk management; systematic review

**INTRODUCTION**

Intravenous drug delivery is a complex process involving multiple possibilities for error. ^1,2^ Because of the immediate therapeutic effect and high bioavailability, intravenous administration routes are widely used in hospitals and especially in critical care settings, such as intensive care units and emergency departments. However, many intravenously administered drugs are high-alert medications, bearing a heightened risk of causing significant patient harm if used in error. ^3^ Intravenously-administered drugs are associated with the highest medication error frequencies and more serious consequences to the patient than any other administration route. ^4–6^ A meta-analysis of observational studies from the United Kingdom demonstrated that administration errors are as much as five times more likely when an intravenous route is used. ^7^ Recent observational multisite studies conducted in the United States and the United Kingdom have reported a high prevalence of intravenous infusion administration errors and procedural failures, even with the use of smart pumps; yet few potentially harmful errors. ^8,9^

To ensure medication safety, effective interventions that can eliminate errors in the intravenous drug delivery process are needed. In healthcare the framework of a just culture ensures balanced accountability for both individuals and the organization responsible for designing and improving systems in the workplace. ^10^ From an organizational point of view, it is essential to identify weaknesses of the current practices and develop systemic defenses to prevent errors reaching patients. ^11,12^ Currently many systemic defenses involve technology-based solutions, and in-hospital drug delivery processes have developed toward closed loop medication management. Closed loop systems consist of electronic prescribing, dispensing of bar-coded unit-dose drugs, safe storage in automated dispensing cabinets, barcode scanning to confirm drug and patient identity, electronic administration records, and clinical decision systems supporting every process step from prescribing to treatment monitoring. ^13–17^ While most intravenous medication errors happen in the administration stage, ^6,18,19^ smart infusion pumps utilizing dose error-reduction software are an essential part of intravenous closed loop systems. ^20^ However, other types of systemic defenses are also needed to ensure intravenous medication safety, such as the use of oral syringes that do not fit to intravenous lines to prevent inadvertent intravenous administration of oral solutions. ^21^

To the best of our knowledge, the systemic defenses related to intravenous medication processes have not been systematically reviewed before. Previous systematic reviews have focused on error prevention strategies in general (e.g., interventions to reduce medication errors in adult ^22^ and pediatric ^23^ intensive care), or one systemic defense (e.g., smart infusion pumps ^24^). Although medication safety is a global priority, systemic defenses related to certain administration routes are not clearly described in many countries. The aim of our study was to explore recent evidence of systemic defenses and their ability to prevent intravenous medication errors in order to inform interprofessional medication safety activities in hospitals.

**METHODS**

**Study design**

A systematic review of recent evidence on systemic defenses aiming to prevent intravenous medication errors in hospitals was carried out following the PRISMA guidelines for undertaking and presenting systematic reviews. ^25^ The quality of the included studies was assessed according to the GRADE system. ^26^ The included articles were analyzed using qualitative content analysis. ^27,28^

**Search strategy**

A systematic literature search was performed in June 2016 on MEDLINE (Ovid), Scopus, Cinahl, and EBM reviews covering the period from January 2005 to June 2016. This period was chosen in order to focus on the most recent evidence published in peer-reviewed journals. An example of the search strategy is presented in Table 1.

We divided the search terms into two themes (‘intravenous medication therapy’ and ‘medication errors’), both of which needed to appear in the included articles. The theme ‘medication error’ was chosen according to our study objectives to explore preventable adverse drug events, which occur as a consequence of errors in the medication process caused by omissions or commissions. ^6,29^ ﻿The search strategy was completed with other terms similar to ‘medication error’ (Table 1), as inconsistency in terminology and definitions related to medication errors is widely known. ^30^ A combination of the themes ‘adverse drug event’ and ‘intravenous’ was also considered. It was not included in the final search strategy, because the combination resulted in a significantly large number of citations with an emphasis on drug safety and adverse drug reactions without objectives relating to medication safety and the medication use-process. We supplemented the search with a manual search of the reference lists of the included articles to identify all relevant publications.

**Inclusion and exclusion criteria**

We applied a predetermined PICO-tool (participants, interventions, comparison, and outcomes) to select studies for inclusion. ^25^ A study was included if participants were hospitalized patients or the study utilized a patient scenario in a simulated hospital environment and patients received intravenous medication. We decided to include simulation studies, because clinical simulation enables assessment of new systemic defenses in safe and controlled environment without risk for patient harm. ^31^ We excluded studies conducted in ambulatory settings, such as home infusion chemotherapy, as we wanted to focus on in-hospital intravenous medication processes. We also excluded studies focusing on multiple administration routes, if the findings related to intravenous administration could not be reliably identified and extracted from the results. Comparison was not required, which means we included studies utilizing both controlled and uncontrolled study designs. Studies applying measures associated with the assessment of systemic defenses intended to prevent intravenous medication errors and/or systemic causes resulting in medication errors were included. Studies exploring unpreventable adverse drug events or only incidence and types of medication errors were excluded. Only English language articles were included. Peer-reviewed journal articles utilizing all methods and study designs were included.

**Study selection**

After the removal of duplicates, the search produced 1,417 potentially relevant publications (Figure 1). Two reviewers (SK, IN) independently selected studies based on the titles. In case of disagreement, the article was included in the next phase, in which the reviewers (SK, IN) independently selected studies based on the abstracts. Disagreements were resolved through discussion and consensus with a third reviewer (A-RH). The reviewers (SK, IN) independently selected studies based on full-texts of the remaining publications. The articles fulfilling the inclusion criteria of both reviewers were included (n=36). Disagreements were resolved through discussion and consensus with the third reviewer (A-RH), which led to the inclusion of nine more articles. A total of 45 publications met the inclusion criteria. Following this, reference lists of the included articles were searched manually for relevant articles (n=12), giving us a total of 57 included studies.

We identified two major themes among the selected articles: systemic defenses aiming to prevent errors and systemic causes of in-hospital intravenous medication errors (Figure 1). The articles focusing on systemic defenses for preventing intravenous medication errors (n=46) are reported in this publication. Articles focusing on systemic causes of intravenous medication errors are discussed in another publication.

**Data extraction and analysis**

Data extraction and analysis were carried out by two of the authors (SK, IN), and the results were carefully reviewed by the other authors (A-RH, MA). Study characteristics, country, study design, setting, evidence quality, systemic defense and comparison, number of patients (or other), primary measures, and key findings were extracted to a table (Supplementary file 1, http://links.lww.com/JPS/A280). We assessed the quality of the evidence using the GRADE system, which has four levels of evidence quality: very low, low, moderate, and high. ^26^ Evidence from randomized controlled trials (RCT) and systematic reviews was graded as high quality and evidence that included observational data was graded as low quality. For example, observational studies conducted in a simulated environment with a small sample size were graded as low quality. Factors which decreased the quality of evidence (e.g., study limitations and inconsistency of results) or increased the quality of evidence (e.g., large magnitude of effect such as a large sample size, controlled study design, and multiple data collection methods and sources such as smart pump-produced log reports, chart reviews, staff reports, and incident reports) were also taken into account. Primary measures used in the articles concerning systemic defenses for preventing intravenous medication errors were extracted to Table 2 to demonstrate methodological variation between the included studies.

We analyzed the included articles using qualitative content analysis to identify systemic defenses and their ability to prevent intravenous medication errors. ^27,28^ The findings were extracted and classified according to which medication process stage was most affected by the systemic defense mechanism (Tables 3 and 4). The systemic defenses, evidence quality, and key findings are presented in Table 3. We assessed the statistical significance of the key findings according to possible statistical analysis presented in the articles, such as *P* value (*P* < .05) and confidence interval (95% confidence interval excludes the null value). Key conclusions and recommendations presented by the authors were extracted to Table 4.

**RESULTS**

**Characteristics and main outcomes of included studies (n=46)**

Our systematic review is based on 46 peer-reviewed original articles (Supplementary file 1, http://links.lww.com/JPS/A280). ^24,32–76^ The studies were conducted in 11 countries, which included the United States (n=22), ^24,32–45,54,65,72–76^ Canada (n=8), ^46–53^ Germany (n=4), ^55–58^ the United Kingdom (n=3), ^59–61^ New Zealand (n=2), ^62,63^ Spain (n=2), ^64,66^ Australia (n=1), ^67^ Brazil and the United States (n=1), ^68^ France (n=1), ^69^ Israel (n=1), ^70^ and Korea (n=1). ^71^ Altogether, 30 studies (65%) were carried out in North America. Most of the studies were conducted in a hospital setting (n=34), ^32–47,51–53,55–58,60–64,66–70,76^ and some in simulated hospital environments (n=11). ^48–50,54,59,65,71–75^ One study was a systematic review including studies conducted both in a hospital setting and a simulated hospital environment. ^24^

There was a lot of variation between the study designs and the evidence quality of the studies. Of the 46 included articles, 38 (83%) ^32–40,43,46–57,59–65,67,69–76^ involved a controlled study design. Only two studies (4%) were graded as high quality: of these two, one applied an RCT design ^62^ and the other was a systematic review ^24^. Six studies (13%) ^32,39,43,46,47,63^ utilized a controlled observational study design with large magnitude of effect, which is why they were graded as moderate quality. Four of these were analyses of incident reports, medication error reports or adverse drug event data, ^32,39,47,63^ and two were observational reviews of patient records. ^43,46^ The remaining 38 studies (83%) ^33–38,40–42,44,45,48–61,64–76^ applied an observational study design without large magnitude of effect, which is why they were graded as low quality. Controlled low quality studies (n=31) applied variable designs: simulation studies (n=11), ^48–50,54,59,65,71–75^ observational reviews of drug charts, medication orders or patient records (n=11), ^33–37,51,52,55,56,60,76^ studies combining multiple methods (n=4), ^61,67,69,70^ analyses of medication error or adverse drug event data (n=3), ^38,40,53^ and analyses of infusion concentrations (n=2). ^57,64^ Some low quality studies (n=7) ^41,42,44,45,58,66,68^ utilized an uncontrolled study design. The study limitations were not reported and their influence was not assessed in five studies (11%). ^45,47,60,68,69^

The primary measures used in the included studies varied, but we identified some shared measures (Table 2). The measure most widely used to assess the effectiveness of a systemic defense was incidence of medication errors, which appeared in 25 studies (54%). ^34,36,38,40,41,46–48,50,52–54,57,59,60,62–65,70–74,76^ There was a lot of variation between the error detection methods used, which makes it difficult to compare results between the studies. Measures quite similar to medication errors, such as adverse drug events and clinical incidents (n=5), ^39,43,63,67,69^ potentially prevented medication errors (n=4), ^42,45,58,66^ and serious medication errors (n=2) ^32,73^ were used in 11 studies (24%). Time to task completion appeared in 12 studies, ^40,54,59,65,69–76^ and it was a commonly-used measure especially in simulation studies (n=9). ^54,59,65,71–75^

**Systemic defenses and their ability to prevent intravenous medication errors**

Systemic defenses, their ability to prevent intravenous medication errors, and statistical significance of the key findings are presented in Table 3. Key conclusions of the included studies and recommendations presented by the authors are presented in Table 4. Of the systemic defenses identified, a majority was related to administration (n=24 studies; 52%), ^24,32,36,38,42,45,48–50,53–56,58,61–63,65,66,68,69,71,73–75^ followed by prescribing (n=8; 18%), ^34,37,43,46,52,60,70,76^ preparation (n=6; 13%), ^40,41,44,57,59,64^ treatment monitoring (n=2; 4%), ^33,35^ and dispensing (n=1; 2%). ^74^ Five studies ^39,47,51,67,72^ (11%) focused on high-risk process standardization, and involved implementation of systemic defenses related to multiple drug delivery process stages.

Systemic defenses, including features of closed loop medication management systems, appeared in 61% of the studies (n=28) (Figure 2), ^24,32–42,45,49,53,54,57,58,60–63,66,69,70,72,74,75^ smart pumps being the systemic defense most widely studied (n=11; 24%). ^24,32,36,38,42,45,49,53,58,66,69^ Besides preventing prescribing errors, computerized orders and decision support systems were found to contribute toward safe dispensing, ^74^ administration, ^54,72,75^ and treatment monitoring ^33,35,72^ by preventing errors related to interpretation of orders, calculation tasks and follow up. In addition to systemic defenses related to closed loop medication management systems, prefilled syringes ^59,73^ and color-coded systems ^65,71,73^ were found to reduce errors in high-risk environments and situations, such as operating rooms and resuscitation.

Even though smart infusion pumps were the systemic defense most widely studied, their effectiveness in medication error prevention remains unclear (Tables 3 and 4). The key component of smart pump is a drug library containing predefined parameters for the drug type, strength, and dosing limits of specific drugs. Soft limits are alerts that can be overridden by clinicians, while hard limits cannot be overridden. Insufficient compliance in drug library use is problematic, as the systemic defense is not active if drug library is bypassed. ^24,32,58,66^ Another issue is high override rate of soft limits, which unlike hard limits do not require changes to pump programming when patient is at risk to get wrong dose. ^32,36,42,45,49,53,58,69^ Opportunities for improvement include use of hard limits and integrating smart pumps with other systemic defenses, such as barcode readers and computerized physician order entry (CPOE) real-time clinical data (e.g., glucose control, respiratory monitoring). ^24,36,42,45,49,58,66^

**DISCUSSION**

To the best of our knowledge, this is the first systematic review to summarize systemic defenses and their ability to prevent intravenous medication errors in hospitals. We found 46 studies involving variable systemic defenses, study designs and evidence quality. There were two high quality studies and six observational studies with large magnitude of effect. Within the included articles, most studies applied an observational study design without large magnitude of effect and did not provide the most rigorous evidence. Over 50% of the studies focused on administration stage, with smart infusion pumps the most widely-studied systemic defence (n=11). We found a limited number of studies exploring other stages of medication use-process; all of them were observational low or moderate quality studies that did not provide the most rigorous evidence. Systemic defenses involving features related to closed loop medication management systems were explored in 28 out of 46 studies.

According to our findings, smart infusion systems reduce, but do not completely prevent, pump programming errors, ^24,38,42,45,49,58,66,69^ which has also been stated in an earlier systematic review. ^24^ We identified high override rates of soft limits and insufficient compliance in drug library use as key limitations for effectiveness. ^24,32,36,49,53,58,66^ To make smart pumps more effective, and thus prevent pump programming errors, increasing the use of hard limits in the drug libraries is important. ^24,49^ Another area of development is the functionality of smart pumps and drug libraries, as a recent study found differences in smart pump compliance both within and between hospital systems, which might be influenced by pump type and the number of drug library profiles. ^77^ Prevention of errors throughout the intravenous medication process requires integrating smart pumps into closed loop medication management systems, such as electronic patient records, ﻿clinical pharmacist’s review of orders, automated compounding systems, barcode verification at the bedside, and real-time clinical monitoring data. ^20,24,36,40–42,45,49,57–59,66,78^

A significant error reduction was reached in one of the high quality studies, which was an RCT study exploring a system designed to reduce errors in the recording and administration of drugs in anesthesia. ^62^ The same system was also studied in another study included in our systematic review, and it involved drug trays and a drug trolley, pre-filled syringes, color-coded labels, barcode drug verification and administration records, and safety alarms to support safe drug administration. ^62,63^ Color-coded systems ^65,71,73^ and prefilled syringes ^59,73^ also showed effectiveness in other studies by reducing errors and time to medication administration in simulated emergency situations. In the future, it is important to ensure the availability of barcoded unit-dose medications to simplify the intravenous drug delivery process in the clinical area. In many countries bar-coded unit dose medications are not yet commercially available and most of the intravenous drug preparation is carried out by nurses and pharmacists in the ward environment, where errors are more likely to happen. ^2,79,80^

Five of the included studies ^39,47,51,67,72^ focused on high-risk medication process standardization and involved systemic defenses in multiple stages of the drug delivery process, which is what the Institute for Safe Medication Practices (ISMP) recommends to support resolving medication safety issues related to high alert medications. ^3^ Another reason to study larger parts of the drug delivery process is to find out how different systemic defenses work together, and on the other hand, how one systemic defense can affect multiple process stages. As an example, in addition to preventing prescribing errors, computerized orders and decision support systems were found to contribute to safe dispensing, administration, and treatment monitoring by preventing errors related to interpretation of orders, calculation tasks, and treatment monitoring. ^33,35,54,72,74,75^

Our study was conducted in accordance with the PRISMA checklist. ^25^ We included only peer-reviewed articles in the analysis and the quality of selected studies was assessed using the GRADE system. ^26^ In addition, we extracted and evaluated the statistical significance of the results presented in the included studies. The literature search was restricted to articles published in English; thus, studies published in other languages were excluded. However, our study indicated that the majority of current evidence in this research area has been published in English-speaking countries, especially in North America.

This systematic review has several limitations. The quality of included studies was relatively low, as most studies (44/46) applied observational methodologies. The studies utilized different measures and study designs, which is why quantitative analysis was not performed. Incidence of medication errors was a commonly-used measure, but there was variation between the error detection methods. None of the studies utilized more than one error detection method, which has been recommended for discovering representative information concerning medication errors. ^81^ Because the data was not summarized statistically, we decided to include an earlier systematic review by Ohashi et al ^24^ to the analysis. If quantitative analysis could have been performed, double-counting the articles included both in our study and systematic review by Ohashi et al ^24^ (n=9) ^32,36,38,39,42,47,49,58,66^ would have been more critical source of bias. Most included studies focused on the administration stage, probably due to administration being the most error-prone stage of the intravenous medication process. ^6,18,19^ The number of studies covering other medication use-process stages was limited, which might be due to the fact that the studies exploring other phases might involve multiple administration routes. As an example, none of the included studies explored automated drug distribution systems, which have been indicated to improve medication safety. ^16^

We had to exclude some promising articles as they appeared to be descriptive project reports and lacked a scientific study design, which might indicate that this research area is still under development. This is why our decision to study systemic defenses in all hospital environments was a good choice, as many defenses can be modified and applied in different care settings. An interesting area for further studies is to explore systemic defenses related to intravenous medication in certain care environments, medical specialties and patient groups. 11 studies were conducted in simulated environment, and it is important to examine these defenses in real life as well. Future studies should explore combinations of systemic defenses and their effectiveness in error prevention in multiple stages of the drug delivery process. As new technology is implemented and more data is available from the systems, it is essential to use this information to assess the effectiveness and areas of development. There is also a need to explore systemic defenses in other settings than inpatient care, while intravenous administration is increasingly common in ambulatory settings.

**CONCLUSIONS**

Most included studies focused on the administration stage, with smart infusion pumps being the most widely-studied systemic defense. We also found a limited number of studies exploring other stages of the medication use-process. Most of the systemic defenses involved features related to closed loop medication management systems. Our study provides healthcare organizations with preliminary knowledge about systemic defenses intended to prevent intravenous medication errors, but more rigorous evidence is needed. There is a need for further studies to explore combinations of systemic defenses and their effectiveness in error prevention.

**ACKNOWLEDGEMENTS**

Sini Kuitunen received a PhD research grant from the University Pharmacy (Yliopiston Apteekki), which is a pharmacy corporation owned by the University of Helsinki. The funder had no input in the design, methods, data collection, analysis, or preparation of this paper.

**REFERENCES**

1. Taxis K, Barber N. Causes of intravenous medication errors: an ethnographic study. *Qual Saf Heal Care*. 2003;12(5):343-347.

2. Mcdowell SE, Mt-isa S, Ashby D, et al. Where errors occur in the preparation and administration of intravenous medicines : a systematic review and Bayesian analysis. *Qual Saf Heal Care*. 2010;19(4):341-345.

3. Institute for Safe Medication Practices. ISMP list of High-Alert Medications in Acute Care Settings. http://www.ismp.org/tools/highalertmedications.pdf. Published 2018. Accessed November 30, 2018.

4. Westbrook JI, Rob MI, Woods A, et al. Errors in the administration of intravenous medications in hospital and the role of correct procedures and nurse experience. *BMJ Qual Saf*. 2011;20(12):1027-1034.

5. Keers RN, Williams SD, Cooke J, et al. Causes of medication administration errors in hospitals: A systematic review of quantitative and qualitative evidence. *Drug Saf*. 2013;36(11):1045-1067.

6. Krähenbühl-Melcher A, Schlienger R, Lampert M, et al. Drug-related problems in hospitals: a review of the recent literature. *Drug Saf*. 2007;30(5):379-407.

7. Mcleod MC, Barber N, Franklin BD. Methodological variations and their effects on reported medication administration error rates. *BMJ Qual Saf*. 2013;22(4):278-289.

8. Schnock KO, Dykes PC, Albert J, et al. The frequency of intravenous medication administration errors related to smart infusion pumps: A multihospital observational study. *BMJ Qual Saf*. 2017;26(2):131-140.

9. Lyons I, Furniss D, Blandford A, et al. Errors and discrepancies in the administration of intravenous infusions: A mixed methods multihospital observational study. *BMJ Qual Saf*. 2018;27(11):892-901.

10. Boysen PG. Just culture: a foundation for balanced accountability and patient safety. *Ochsner J*. 2013;13(3):400-406.

11. Reason J. Human error: models and management. *Bmj*. 2000;320(March):768-770.

12. Yip L, Farmer B. High Reliability Organizations—Medication Safety. *J Med Toxicol*. 2015;11(2):257-261.

13. Franklin BD, O’Grady K, Donyai P, et al. The impact of a closed-loop electronic prescribing and administration system on prescribing errors, administration errors and staff time: A before-and-after study. *Qual Saf Heal Care*. 2007;16(4):279-284.

14. Austin JA, Smith IR, Tariq A. The impact of closed-loop electronic medication management on time to first dose: a comparative study between paper and digital hospital environments. *Int J Pharm Pract*. 2018.

15. Hwang Y, Yoon D, Ahn EK, et al. Provider risk factors for medication administration error alerts: analyses of a large-scale closed-loop medication administration system using RFID and barcode. *Pharmacoepidemiol Drug Saf*. 2016;25:1387-1396.

16. Ahtiainen HK, Kallio MM, Airaksinen M, et al. Safety, time and cost evaluation of automated and semi-automated drug distribution systems in hospitals: A systematic review. *Eur J Hosp Pharm* . 2019:1-10.

17. Young J, Slebodnik M, Sands L. Bar Code Technology and Medication Administration Error. *J Patient Saf*. 2010;6(2):115-120.

18. Valentin A, Capuzzo M, Guidet B, et al. Errors in administration of parenteral drugs in intensive care. *BMJ*. 2009;338.

19. Thomas AN, Macdonald JJ. A review of patient safety incidents reported as ‘severe’ or ‘death’ from critical care units in England and Wales between 2004 and 2014. *Anaesthesia*. 2016;71:1013-1023.

20. Trbovich P, Easty A. *Smart Pump Implementation: A Guide for Healthcare Institutions*.; 2012. http://s3.amazonaws.com/rdcms-aami/files/production/public/FileDownloads/Foundation/SafetyInnovation/2012_SI_Smart_Pump_Implementation.pdf.

21. Grissinger M. Oral Syringes : Making Better Use of a Crucial And Economical Risk-Reduction Strategy. *P&T*. 2013;38(1):5-6.

22. Manias E, Williams A, Liew D. Interventions to reduce medication errors in adult intensive care : a systematic review. *Br J Clin Pharmacol*. 2012;74(October 2011).

23. Manias E, Kinney S, Cranswick N, et al. Interventions to Reduce Medication Errors in Pediatric Intensive Care. *Ann Pharmacother*. 2014;48(10):1313-1331.

24. Ohashi K, Dalleur O, Dykes PC, et al. Benefits and Risks of Using Smart Pumps to Reduce Medication Error Rates: A Systematic Review. *Drug Saf*. 2014;37(12):1011-1020.

25. Moher D, Shamseer L, Clarke M, Ghersi D, Liberati A, Petticrew M, Shekelle P SL. Preferred Reporting Items for Systematic Review and Meta-Analysis Protocols (PRISMA-P) 2015 statement. *Syst Rev*. 2015;4(1):1-9.

26. Guyatt GH, Oxman AD, Kunz R, et al. GRADE: What is “Quality of evidence” and why is it important to clinicians? *BMJ*. 2008;336:995-998.

27. Elo S, Kyngäs H. The qualitative content analysis process. *J Adv Nurs*. 2008;62(1):107-115.

28. Hsieh H-F, Shannon SE. Three Approaches to Qualitative Content Analysis. *Qual Healthc Res*. 2005;15(9):1277-1288.

29. National Coordinating Council for Medication Error Reporting and Prevention. About Medication Errors. https://www.nccmerp.org/about-medication-errors. Accessed November 28, 2018.

30. Lisby M, Nielsen LP, Brock B, et al. How are medication errors defined? A systematic literature review of definitions and characteristics. *Int J Qual Heal Care*. 2010;22(6):507-518.

31. Lamé G, Dixon-Woods M. Using clinical simulation to study how to improve quality and safety in healthcare. *BMJ Simul Technol Enhanc Learn*. 2018:bmjstel-2018-000370.

32. Rothschild JM, Keohane CA, Cook EF, et al. A controlled trial of smart infusion pumps to improve medication safety in critically ill patients. *Crit Care Med*. 2005;33(3):533-540.

33. Boord JB, Sharifi M, Greevy RA, et al. Computer-based Insulin Infusion Protocol Improves Glycemia Control over Manual Protocol. *J Am Med Informatics Assoc*. 2007;14(3):278-287.

34. Lehmann CU, Kim GR, Gujral R, et al. Decreasing errors in pediatric continuous intravenous infusions. *Pediatr Crit Care Med*. 2006;7(3):225-230.

35. Muzyk AJ, Rivelli SK, Jiang W, et al. A computerized physician order entry set designed to improve safety of intravenous haloperidol utilization. A Retrospective Study in Agitated Hospitalized Patients. *Drug Saf*. 2012;35(9):725-731.

36. Nuckols TK, Bower AG, Paddock SM, et al. Programmable infusion pumps in ICUS: An analysis of corresponding adverse drug events. *J Gen Intern Med*. 2008;23(1 SUPPL.):41-45.

37. Pell JM, Cheung D, Jones MA, et al. Don’t fuel the fire: Decreasing intravenous haloperidol use in high risk patients via a customized electronic alert. *J Am Med Informatics Assoc*. 2014;21(6):1109-1112.

38. Larsen GY, Parker HP, Cash J, et al. Standard Drug Concentrations and Smart-Pump Technology Reduce Continuous-Medication-Infusion Errors in Pediatric Patients. *Pediatrics*. 2005;116(1):e21-e25.

39. Prewitt J, Schneider S, Horvath M, et al. PCA Safety Data Review After Clinical Decision Support and Smart Pump Technology Implementation Introduction. *J Patient Saf*. 2013;9(2):103-109.

40. Reece KM, Lozano MA, Roux R, et al. Implementation and evaluation of a gravimetric i.v. workflow software system in an oncology ambulatory care pharmacy. *Am J Heal Pharm*. 2016;73(3):165-173.

41. Deng Y, Lin AC, Hingl J, et al. Risk factors for i . v . compounding errors when using an automated workflow management system. *Am J Heal Pharm*. 2016;73(12):887-894.

42. Evans RS, Carlson R, Johnson K V., et al. Enhanced notification of infusion pump programming errors. *Stud Health Technol Inform*. 2010;160(PART 1):734-738.

43. White JRM, Veltri MA, Fackler JC. Preventing adverse events in the pediatric intensive care unit: Prospectively targeting factors that lead to intravenous potassium chloride order errors. *Pediatr Crit Care Med*. 2005;6(1):25-31.

44. Kaakeh Y, Phan H, DeSmet BD, et al. Enhanced photoemission spectroscopy for verification of high-risk i.v. medications. *Am J Heal Pharm*. 2008;65(1):49-54.

45. Williams CK, Maddox RR, Heape E, et al. Application of the IV medication harm index to assess the nature of harm averted by “smart” infusion safety systems. *J Patient Saf*. 2006;2(3):132-139.

46. Larose G, Bailey B, Lebel D. Quality of orders for medication in the resuscitation room of a pediatric emergency department. *Pediatr Emerg Care*. 2008;24(9):609-614.

47. Paul JE, Bertram B, Antoni K, et al. Impact of a comprehensive safety initiative on patient-controlled analgesia errors. *Anesthesiology*. 2010;113(6):1427-1432.

48. Prakash V, Koczmara C, Savage P, et al. Mitigating errors caused by interruptions during medication verification and administration: Interventions in a simulated ambulatory chemotherapy setting. *BMJ Qual Saf*. 2014;23(11):884-892.

49. Trbovich PL, Pinkney S, Cafazzo JA, et al. The impact of traditional and smart pump infusion technology on nurse medication administration performance in a simulated inpatient unit. *Qual Saf Heal Care*. 2010;19(5):430-434.

50. White RE, Trbovich PL, Easty AC, et al. Checking it twice: An evaluation of checklists for detecting medication errors at the bedside using a chemotherapy model. *Qual Saf Heal Care*. 2010;19(6):562-567.

51. Dubrofsky L, Kerzner RS, Delaunay C, et al. Interdisciplinary Systems-Based Intervention to Improve IV Hydration during Parenteral Administration of Acyclovir. *Can J Hosp Pharm*. 2016;69(1):7-13.

52. Kaplan GG, Bates D, Mcdonald D, et al. Inappropriate use of intravenous pantoprazole: Extent of the problem and successful solutions. *Clin Gastroenterol Hepatol*. 2005;3(12):1207-1214.

53. Guérin A, Tourel J, Delage E, et al. Accidents and Incidents Related to Intravenous Drug Administration: A Pre–Post Study Following Implementation of Smart Pumps in a Teaching Hospital. *Drug Saf*. 2015;38(8):729-736.

54. Deshpande GG, Torres Jr. A, Buchanan DL, et al. Computerized pharmaceutical algorithm reduces medication administration errors during simulated resuscitations. *J Pediatr Pharmacol Ther*. 2010;15(4):274-281.

55. Bertsche T, Mayer Y, Stahl R, et al. Prevention of intravenous drug incompatibilities in an intensive care unit. *Am J Heal Pharm*. 2008;65(19):1834-1840.

56. Bertsche T, Veith C, Stahl A, et al. A purging procedure for pantoprazole and 4-lumen catheters to prevent IV drug incompatibilities. *Pharm World Sci*. 2010;32(5):663-669.

57. Dehmel C, Braune SA, Kreymann G, et al. Do centrally pre-prepared solutions achieve more reliable drug concentrations than solutions prepared on the ward? *Intensive Care Med*. 2011;37(8):1311-1316.

58. Kastrup M, Balzer F, Volk T, et al. Analysis of Event Logs from Syringe Pumps. A Retrospective Pilot Study to Assess Possible Effects of Syringe Pumps on Safety in a University Hospital Critical Care Unit in Germany. *Drug Saf*. 2012;35(7):563-574.

59. Adapa RM, Mani V, Murray LJ, et al. Errors during the preparation of drug infusions: A randomized controlled trial. *Br J Anaesth*. 2012;109(5):729-734.

60. Manjaly JG, Reece-Smith AM, Sivaloganathan SS, et al. Improving dosing of gentamicin in the obese patient: a 3-cycle drug chart and case note audit. *JRSM Short Rep*. 2012;3(4):1-12.

61. Evley R, Russell J, Mathew D, et al. Confirming the drugs administered during anaesthesia: A feasibility study in the pilot National Health Service sites, UK. *Br J Anaesth*. 2010;105(3):289-296.

62. Merry AF, Webster CS, Hannam J, et al. Multimodal system designed to reduce errors in recording and administration of drugs in anaesthesia: Prospective randomised clinical evaluation. *BMJ*. 2011;343(7826):1-14.

63. Webster CS, Larsson L, Frampton CM, et al. Clinical assessment of a new anaesthetic drug administration system: A prospective, controlled, longitudinal incident monitoring study: ORIGINAL ARTICLE. *Anaesthesia*. 2010;65(5):490-499.

64. Campino A, Santesteban E, Pascual P, et al. Strategies implementation to reduce medicine preparation error rate in neonatal intensive care units. *Eur J Pediatr*. 2016;175(6):755-765.

65. Feleke R, Kalynych CJ, Lundblom B, et al. Color coded medication safety system reduces community pediatric emergency nursing medication errors. *J Patient Saf*. 2009;5(2):79-85.

66. Manrique-Rodríguez S, Sánchez-Galindo AC, López-Herce J, et al. Impact of implementing smart infusion pumps in a pediatric intensive care unit. *Am J Heal Pharm*. 2013;70(21):1897-1906.

67. Barras M, Moore D, Pocock D, et al. Reducing the risk of harm from intravenous potassium: A multi-factorial approach in the haematology setting. *J Oncol Pharm Pract*. 2014;20(5):323-331.

68. Chanes DC, Pedreira M da LG, De Gutiérrez MGR. Antineoplastic agents extravasation from peripheral intravenous line in children: A simple strategy for a safer nursing care. *Eur J Oncol Nurs*. 2012;16(1):17-25.

69. Cour M, Hernu R, Bénet T, et al. Benefits of smart pumps for automated changeovers of vasoactive drug infusion pumps: A quasi-experimental study. *Br J Anaesth*. 2013;111(5):818-824.

70. Vardi A, Efrati O, Levin I, et al. Prevention of potential errors in resuscitation medications orders by means of a computerised physician order entry in paediatric critical care. *Resuscitation*. 2007;73(3):400-406.

71. Cho J, Chung HS, Hong SH. Improving the safety of continuously infused fluids in the emergency department. *Int J Nurs Pract*. 2013;19(1):95-100.

72. Lee A, Faddoul B, Sowan A, et al. Computerisation of a paper-based intravenous insulin protocol reduces errors in a prospective crossover simulated tight glycaemic control study. *Intensive Crit Care Nurs*. 2010;26(3):161-168.

73. Moreira ME, Hernandez C, Stevens AD, et al. Color-coded prefilled medication syringes decrease time to delivery and dosing error in simulated emergency department pediatric resuscitations. *Ann Emerg Med*. 2015;66(2):97-106.

74. Sowan AK, Vaidya VU, Soeken KL, et al. Computerized Orders with Standardized Concentrations Decrease Dispensing Errors of Continuous Infusion Medications for Pediatrics. *J Pediatr Pharmacol Ther*. 2010;15(3):189-202.

75. Sowan AK, Gaffoor MI, Soeken K, et al. Impact of Computerized Orders for Pediatric Continuous Drug Infusions on Detecting Infusion Pump Programming Errors: A Simulated Study. *J Pediatr Nurs*. 2010;25(2):108-118.

76. Bailey AM, Stephan M, Weant KA, et al. Dosing of Appropriate Antibiotics and Time to Administration of First Doses in the Pediatric Emergency Department. *J Pediatr Pharmacol Ther*. 2015;20(4):309-315.

77. Giuliano KK, Su WT, Degnan DD, et al. Intravenous Smart Pump Drug Library Compliance: A Descriptive Study of 44 Hospitals. *J Patient Saf*. 2018;14(4):e76-e82.

78. Wiseman ML, Poole S, Ahlin A, et al. Reducing intravenous infusion errors: an observational study of 16 866 patients over five years. *J Pharm Pract Res*. 2018;48(1):49-55.

79. Aguado-Lorenzo V, Weeks K, Tunstell P, et al. Accuracy of the concentration of morphine infusions prepared for patients in a neonatal intensive care unit. *Arch Dis Child*. 2013;98(12):975-979.

80. Campino A, Arranz C, Unceta M, et al. Medicine preparation errors in ten Spanish neonatal intensive care units. *Eur J Pediatr*. 2016;175(2):203-210.

81. Härkänen M, Turunen H, Vehviläinen-Julkunen K. Differences Between Methods of Detecting Medication Errors: A Secondary Analysis of Medication Administration Errors Using Incident Reports, the Global Trigger Tool Method, and Observations. *J Patient Saf*. 2016;00(00):1-9.

Figure 1. Flowchart of study.

Figure 2. Systemic defenses related to closed loop medication management explored in the included studies (n=46) (modified ^13–16,20^).

Table 1. Search strategy for MEDLINE (Ovid).

Table 2. Synthesis of the primary measures used in the included studies (n=46).

Table 3. Systemic defenses, evidence quality (L=low, M=moderate, H=high), key findings, and statistical significance of the findings in the included studies (n=46).

Table 4. Conclusions and recommendations presented by the authors of the included studies (n=46).
