# Supplementary material for: Systemic Defenses to Prevent Intravenous Medication Errors in Hospitals: A Systematic Review
Source: J Patient Saf. 2020 Mar 17;17(8):e1669–80. doi: 10.1097/PTS.0000000000000688 (PMC8612901; doi:10.1097/PTS.0000000000000688)
Supplement: SUPPLEMENTARY MATERIAL [file pts-17-e1669-s003.docx]

**Supplementary file 1. Overview of the studies included in the systematic review (n=46).**

| **Reference and country** | **Study design (evidence quality) and setting** | **Systemic defense (comparison)** | **Number of patients (or other) (n)** | **Primary measures** | **Key findings** |
| --- | --- | --- | --- | --- | --- |
| **HIGH EVIDENCE QUAILTY (n=2): randomized controlled trial (RCT) (n=1), systematic review (n=1)** | | | | | |
| **Randomized controlled trial (RCT) (n=1)** | | | | | |
| Merry ym. 2011 [62]  New Zealand | Prospective randomized controlled open label clinical trial (High), 5 operating theaters in a major tertiary referral hospital | SAFERSleep system: customized drug trays and trolley, pre-filled syringes, color-coded labels, barcode drug verification and administration record, safety alarms (vs. conventional practice with a manual anesthetic record) | 1,244 in total; 613 intervention and 631 control | 1) Incidence of medication errors ﻿detected by direct observation and reconciliation of the used drug vials against recorded administrations  2) Lapses in responding to an intermittent visual stimulus (vigilance latency task) | 1) The overall mean rate of errors per 100 administrations was 9.1 with SAFERSleep system (95% CI 6.9–11.4; 1/11 administrations) and 11.6 with conventional methods (9.3–13.9; 1/9) (*P* = .045). Most were recording errors. Error rates were lower when anesthetists applied key principles of the new system (scanning the drug barcode before administration and keeping the voice prompt active) than when they did not: mean 6.0 (3.1–8.8) errors per 100 administrations vs. 9.7 (8.4–11.1) respectively  (*P* = .004).  2) Lapses in vigilance latency task occurred in 12% (58/471) of cases with SAFERSleep and 9% (40/473) with conventional methods (*P* = .052). |
| **Systematic review (n=1)** | | | | | |
| Ohashi ym. 2014 [24]  United States | Systematic review (High), hospital setting or a simulated hospital environment | Smart infusion pumps (comparison not required) | 21 (studies included in the analysis; 16/21 studies were conducted in the USA and others in Canada, Australia, Spain, and Germany) | 1) benefits of smart infusion pumps  2) negative effects of smart infusion pumps | ﻿1) Interception and prevention of various error types such as wrong rate, wrong dose, and pump setting errors (5/21 studies). Two studies showed a reduction of adverse drug event rates, while two studies observed no difference. Smart pumps’ capacity to record data about the infusion process was seen as a benefit in 5 studies.  2) Low compliance rates regarding use of smart pump drug library (62-98%), the overriding of soft ﻿alerts, not intercepting error types other than wrong infusion rate, lack of integration with other systems, and the possibility of using the wrong drug library. |
| **MODERATE EVIDENCE QUALITY: controlled observational studies with large magnitude of effect (n=6)** | | | | | |
| Larose et al. 2008 [46]  Canada | Retrospective controlled observational review of patient records (Moderate), Pediatric ED setting (resuscitation and trauma room) | Standard order form for intravenous medications and fluids (vs. before intervention) | 719 in total; 347 intervention period and 372 control (medication orders) | 1) Completeness of written orders  2) Incidence of medication errors  3) Error type  4) Error severity | 1) Complete written orders: 93/281 (33%) in the intervention period and 14/276 (5%) in the control period. Drugs administered to patients without written orders or confirmations of verbal orders: 66 (19%) in the intervention period and 96 (26%) in the control period.  2) There were 20 errors/347 orders (6%) in the intervention period and 55/372 (15%) in the control period.  3) Prescribing errors: intervention period 8/20 (40%) and control period 32/55 (58%). Administration errors: intervention period 23/55 (42%) and control period 12/20 (60%).  4) Most errors had no adverse effects on patients. 11 errors (10 in the control and 1 in the intervention period) required intervention (e.g. need for additional dose because of underdosage). |
| Paul et al. 2010 [47]  Canada | Longitudinal controlled observational incident monitoring study (Moderate), 3 tertiary care hospitals | Patient-controlled analgesia (PCA) safety intervention: new PCA pumps, pre-printed physician orders, nursing and patient education, a manual independent double-check, and a formal nursing transfer of accountability (vs. before intervention) | 25,198 in total;  12,193 after intervention and 13,005 before intervention | 1) Incidence of self-reported PCA medication errors  2) Error type  3) Error causes  4) Error severity  5) The process stage at which the error occurred | 1) 62 errors in total: 49 (79%) before intervention and 13 (21%) after.  2) Incorrect doses in 77.4% of errors (n=48), 12.9% faulty PCA setup errors usually involving improper setup of IV-tubing.  3) The most common causes were pump-programming errors (n=21, 33.9%; all before intervention), orders given by other than acute pain service physicians (14.6%), and inadequate nurse education (12.9%).  4) Errors caused no harm (66%) and some harm (34%). Patient harm included respiratory depression (16%) requiring oxygen and/or naloxone, uncontrolled pain (13%), and sedation (6%).  5) Errors occurred at every stage in the drug-order workflow: prescribing (1.5%), dispensing (1.5%), order transcription (8%), and drug administration (89%). |
| Prewitt et al. 2013 [39]  United States | Retrospective controlled review of adverse drug event data (Low), university hospital setting | ﻿Clinical decision support via CPOE and PCA smart pumps for the prescribing and administration of opioids therapy (vs. before intervention) | 39,224 in total; 18,117 intervention and 21,107 control (PCA days) | Adverse drug events ﻿detected by voluntary reporting (VRS) and automatic surveillance (ADE-S) | ﻿112 ADE-S and 50 VRS events in control period vs. 75 ADE-S and 12 VRS events in intervention period. The ADE-S PCA events per 1,000 patient PCA days went from 5.3 (control) to 4.2 (intervention) (*P* = .09), a decrease of 22.0%. VRS PCA events per 1,000 patient PCA days decreased from 2.4 (control) to 0.66 (intervention) (*P* < .0001), a reduction of 72.0%. There was a difference in the ADE-S data for causality (*P* < .0001) with sleep apnea and renal insufficiency. Voluntary report system safety events were statistically significant for obese (BMI ≥30) and overweight patients. |
| Rothschild et al. 2005 [32]  United States | Observational analysis of medication error data (Moderate),  cardiac surgical intensive care and step-down unit setting | Smart pumps with drug library (including standard concentrations and soft limits; no hard limits) (vs. drug library off) | ﻿8,172 in total; 3,869 intervention and 4,276 control (patient-pump days; 5,295 IV medication in the intervention period and 5,364 in the control period) | 1) serious medication errors (detected from smart-pump  ﻿alert log reports, chart review, staff reports, hospital incident reports, and a computerized ADE surveillance monitor)  2) potentially risky practices | 1) A total of 180 ﻿serious medication errors, including 14 and 11 preventable adverse drug events and 73 and 82 non-intercepted potential adverse drug events in the control and intervention periods. The rates of serious medication errors in the control and intervention periods were 2.03 and 2.41 per 100 patient-pump-days (*P* = .124). ﻿  2) Practice violations during the intervention periods included 571 (25%) bypasses of the drug library. Medications were also frequently administered without documentation of physician orders in both periods (n=823; 7.7%). |
| Webster et al. 2010 [63]  New Zealand | Prospective controlled longitudinal incident monitoring study (Moderate), 2 tertiary care teaching hospitals | Safety-orientated system of delivering parenteral anesthetic drugs: customized drug trays and trolley, pre-filled syringes, color-coded drug labels, barcode scanner for drug verification and administration record, safety alarms (vs. conventional methods) | 59,273 in total;  10,816 with intervention and 63,662 with conventional methods (Hospital A: 22,050 and Hospital B: 41,612)  (incident forms; 79.6% response rate of total 74,478 anesthetics) | 1) Incidence of medication errors detected by self-reporting  2) Error types  3) Incidence of adverse outcomes | 1) Fewer errors with new system than with conventional methods: 58 errors in an estimated 183,852 administrations (0.032%, 95% CI 0.024–0.041%) vs. 268 in 550,105 (0.049%, 95% CI 0.043– 0.055%) respectively  (*P* = .002), a relative reduction of 35% (difference 0.017%, 95% CI 0.006– 0.028%).  2) There were significantly fewer dose and omission errors reported per administration with the use of the new system than with conventional methods. Errors in the ‘other’ category were greater with the new system – a result attributable to the administration of seven pre-filled syringes that had passed their expiration dates. Of substitution errors, a significantly lower proportion of wrong drugs belonged to a different color-code class than the drug intended with the new system than with conventional methods  3) No major adverse outcomes from these errors were reported with the new system while 11 (0.002%) were reported with conventional methods (*P* = .055). |
| White et al. 2005 [43]  United States | Controlled observational review of patient record (Moderate), PICU | A mandatory drug request form for intravenous potassium chloride orders (vs. before intervention) | 1,658 in total;  1,492 before and 166 after intervention (intravenous potassium chloride orders) | 1) Elevated potassium levels after intravenous potassium chloride (over 4.5 mmol/L)  2) Proximal causes of error | 1) The incidence of post-infusion elevations in serum potassium decreased from a rate of 7.7% (103/1 341) before the drug request form to 0% (0/150) after the drug request form (*P* < .001).  2) Proximal causes of error were also reduced. The number of patients with a creatinine >2 mg/dL receiving intravenous potassium chloride decreased from 28.4% to 14.2% (*P* < .001). The number of intravenous potassium chloride infusions administered to patients where serum potassium value was >4.5 mmol/L decreased significantly (2.9% vs. 0.0%, *P* < .02). The incidence rates of both verbal orders and failure to write the order in a correct format were reduced to zero. |
| **LOW EVIDENCE QUALITY: CONTROLLED OBSERVATIONAL STUDY (n=32): simulation study (n=11); review of drug chart, orders or patient record (n=11); controlled analyses of medication error or adverse drug event data (n=4); study combining multiple methods** **(n=4); analyses of infusion concentrations (n=2)** | | | | | |
| **Controlled observational simulation study (n=11)** | | | | | |
| Adapa et al. 2012 [59]  United Kingdom | Randomized, blinded, controlled simulation study  (Low), simulated intensive care unit (ICU) | Using prefilled syringes (vs. preparing drug infusions at the bedside) in emergency situations | 96 in total; 48 intervention and 48 control  (scenarios performed by  48 nurses; 24 intervention and 24 control) | 1) Time taken for the infusion to be started  2) Final concentration of drugs (including comparison between prefilled syringes prepared by doctor, pharmacy and industry) | 1) 156 s when using pre-filled syringes vs. 276 s when preparing infusions at the bedside, a mean delay of 106 s (95% confidence interval (CI) 73–140 s, *P* = .0001).  2) Errors were 17.0 times less likely with pre-filled syringes (95% CI 5.2–55.5). Infusions prepared by pharmacy and industry were more likely to contain the expected concentration (*P* = .001 for norepinephrine and *P* < .001 for epinephrine). One infusion prepared from ampoules contained one-fifth of the expected concentration of epinephrine; another contained none at all. |
| Cho et al. 2013 [71]  Korea | Prospective controlled simulation study (Low), Simulated ED setting | Color-coded label system in ED fluids (vs. before intervention) | 414 in total; 207  intervention and 207 control  (scenarios performed by 69 nurses all acting as both intervention and control) | 1) Time taken for task completion  2) Accuracy of finding the right fluid according to scenario | 1) Time improvements for ED nurses (n=22) from pre- to post-intervention for potassium, heparin and normal saline scenarios were 24.8 vs. 6.9 s (*P* < .001), 17.8 vs. 6.3 s (*P* < .001) and 19.2 vs. 6.3 s (*P* < .001), for ICU nurses (n=23) 22.4 vs. 5.1 s (*P* < .001), 17.8 vs. 5.4 s (*P* < .001) and 18.6 vs. 4.9 s (*P* < .001) and for nursing students (n=24) 58.9 vs. 9.0 s (*P* < .001), 22.5 vs. 7.3 s (*P* < .001) and 32.0 vs. 6.9 s (*P* < .001).  2) Pre-intervention numbers of incorrect fluids were 2 (ED nurses), 2 (ICU nurses) and 9 (students). No incorrect fluids were indicated by participants at post-intervention. |
| Deshpande et al. 2010 [54]  United States | Simulated within-subject crossover-controlled study (Low), Simulated pediatric ED and ICU setting  (resuscitation scenarios) | Pharmaceutical algorithm computerized calculator (pac2): conversion of physician medication orders to correct volumes and administration rates (vs. before intervention) | 66 in total; 33 intervention and 33 control  (scenario sets performed by 33 nurses all acting as both intervention and control) | 1) Drug volumes calculated and drawn accurately (i.e., ± 10% correct volume) into syringes  2) Recall of essential medication information  3) Recognition of unsafe doses  4) Time taken for task completion | 1) Use of the pac2 resulted in a significantly higher percentage of medication volumes calculated and drawn accurately (91% vs. 61%, *P* < .0001).  2) Use of the pac2 resulted in a higher percentage of correct recall of essential medication information (97% vs. 45%, *P* < .0001)  3) Use of the pac2 resulted in better recognition of unsafe doses (93% vs. 19%, *P* < .0001) as compared to usual practice.  4) The pac2 significantly reduced average medication calculation times (1.5 min vs. 1.9 min, *P* = .0028) as compared to usual practice. |
| Feleke et al. 2009 [65]  United States | Controlled observational simulation study (Low), simulated community ED setting | Color-coded pediatric medication safety (CCMS) system for preparation and administration (vs. before intervention) | 320 in total; 160 intervention and 160 control  (scenarios performed by 16 nurses acting as both intervention and control) | 1) time to task completion from order to conversion and administration  2) drug conversion dose in milliliters  3) verbal description of the dilution  4) intended injection or infusion time  5) Ability to detect 10-fold order errors (4 errors in 8 scenarios) | 1) The median time to task completion pre-intervention 109 s (interquartile range, 44–626) and with the CCMS system a median of 28 s (interquartile range, 14–43;  *P* < .001).  2) Pre-intervention 25.6% of medications converted incorrectly compared with 2.5% with CCMS system, a 23% improvement (95% confidence interval [CI], 13–33;  *P* < .001).  3) Pre-intervention 35.6% were diluted incorrectly compared with 0.63%, a 35% improvement (95% CI, 26–44; *P* < .001).  4) Pre-intervention 54.7% administered incorrectly compared with 3.9%, a 51% improvement (95% CI, 39–61; *P* < .001).  5) Only 20% of 10-fold physician order errors were recognized pre-intervention but 93% were recognized using the CCMS system, a 73% improvement. |
| Lee et al. 2010 [72]  United States | Crossover controlled simulation study (Low), simulated ICU environment | Computerized continuous intravenous insulin protocols for tight glycemic control (TGC) (vs. paper-based protocol) | 1,240; 620 intervention and 620 control (scenarios performed by 62 nurses acting as both intervention and control) | 1) Number and types of errors  2) Time to task completion  3) User satisfaction | 1) The number of errors was lower with computerized protocol in insulin initiation (3 vs. 8, *P* = .096), titration (13 vs. 113, *P* = .0001) and transition phases (9 vs. 23,  *P* = .001)  2) Initiation: computerized protocol took longer (1min47s±1min vs. 1min 22s ± 0.5min, *P* = .002). Titration: paper protocol took more time (9 min 32s±3min vs. 6min 5s±2min, *P* = .0001). Transition: no significant difference in completion time (1 min 50 s ± 1.5 min vs. 1 min 21s±1.5min, *P* = .09).  3) Computerized protocols were associated with higher user satisfaction. |
| Moreira et al. 2015 [73]  United States | Prospective, block-randomized, crossover-controlled study (Low), Simulated pediatric ED setting | Prefilled medication syringes labeled with color-coded volumes corresponding to the weight-based dosing of the Broselow Tape (vs. conventional medication administration) | 241 in total; 123 intervention and 118 control  (IV-doses administered in 2 resuscitation scenarios performed by 11 physicians and 10 nurses with and without intervention) | 1) Time from initiation of medication preparation to completed administration  2) Dosing errors  3) Critical dosing errors (10% deviation of the ideal dose) | 1) Median time to delivery of all doses for the conventional and color-coded delivery groups was 47s (95% CI 40–53s) and 19s (95% CI 18–20s), respectively (difference=27s; 95% CI 21–33s).  2) With conventional delivery method, 9/10 teams (90%) made at least 1 dosing error and 31/118 doses administered (26%; 95% CI 19%–35%) were dosing errors. With color-coded method, 4/10 teams (40%) made at least 1 dosing error. 5/123 doses administered (4%; 95% CI 1%–9%) were dosing errors.  3) With the conventional method, incidence of critical dosing errors was 20/118 doses (17%) and 7/10 (70%) teams made 1 or more critical dosing errors. With color-coded method, there were no critical dosing errors in 123 doses administered (difference=17%; 95% CI 4%–530%). |
| Prakash et al. 2014 [48]  Canada | High-fidelity simulation study (observational,  within-subjects and between-subjects controlled) (Low), simulated ambulatory chemotherapy setting | Interventions to reduce errors in verification (verification booth, standardized workflow, speaking aloud) and administration (visual timers for IV pushes, no interruption zones, speaking aloud, reminder signage) when interrupted (vs. interrupted and uninterrupted before intervention) | 110 in total; 38 intervention and 36 + 36 control  (scenarios; 2 scenarios with 7 tasks performed by 18 nurses before and 19 nurses after intervention) | Medication error rate | ﻿Significantly more nurses committed errors when interrupted than when uninterrupted. With use of interventions when interrupted, significantly fewer nurses made errors in verifying medication volumes contained in syringes (16/18; 89% pre-intervention error rate vs. 11/19; 58% post-intervention error rate; *P* = .038; Fisher’s exact test) and programmed in ambulatory pumps (17/18; 94% pre-intervention vs. 11/19; 58% post-intervention;  *P* = .012). The rate of error commission significantly decreased with use of interventions when interrupted during intravenous push (16/18; 89% pre-intervention vs. 6/19; 32% post-intervention; *P* = .017) and pump programming (7/18; 39% pre-intervention vs. 1/19; 5% post-intervention; *P* = .017). No statistically significant differences were observed for other medication verification tasks. |
| Sowan ym. 2010a [74]  United States | Controlled observational simulation study (Low), simulated pediatric hospital pharmacy setting | CPOE generated ﻿orders for continuous infusions with standardized concentrations (vs. handwritten orders ﻿with weight-based (rule-of-six) dosing, and non-standardized concentrations vs. handwritten orders with ﻿deliberate errors) | 140 in total; 50 intervention, 50 control without errors and 40 control with errors (scenarios performed by 10 pharmacists acting as both intervention and controls) | 1) incidence of medication errors detected by observation of infusion labels  2) incidence of high-risk errors (≥100% deviation of order)  3) time to task completion  4) user satisfaction | 1) CPOE eliminated all error types except wrong concentration. With CPOE, 4% of infusions processed contained errors, compared with 26% of the first group of handwritten orders and 45% of the second group of handwritten orders (p<0.03).  2) ﻿There was one CPOE infusion (2%) with high-risk errors, not statistically different from the 5 infusions with high risk errors (10%) in the first handwritten group, or the 4 infusions (10%) in the second handwritten group  (*P* > .05). The majority of high-risk errors in both methods resulted from processing the wrong drug amount.  ﻿3) Using CPOE orders, participants required less processing time per infusion order (2 min, 5 s ± 58 s) compared with time per infusion order in the first handwritten order sheet group (3 min, 7 s ± 1 min, 20 s) and the second handwritten order sheet group (3 min, 26 s ± 1 min, 8 s), (*P* < .01).  4) Pharmacists were more satisfied with CPOE orders compared with the handwritten method (*P* = .0001). |
| Sowan ym. 2010b [75]  United States | Controlled observational simulation study (Low), simulated pediatric hospital setting | CPOE-generated ﻿  orders for continuous infusions with standardized concentrations (vs. handwritten orders) | 216 in total; 108 intervention and 108 control (scenarios performed by 36 nurses acting as both intervention and control) | 1) time to task completion  2) incidence of errors in verification of infusion pump settings detected by direct observation  3) user satisfaction | 1) ﻿Using the computerized orders, nurses were able to check the accuracy of pump settings in less time (6 min 18 s ± 2 min 26 s) as compared with using the handwritten orders (8 min 47 s ± 3 min 6 s; *P* < .0001).  2) ﻿Of the 108 infusions in each group, 72 had been programmed with deliberate errors in the infusion pump settings, whereas 36 were programmed with the correct settings. Using CPOE orders, nurses failed to detect dose or concentration errors in 37% of infusions with errors, as compared with 39% of infusions using the handwritten orders (*P* = .68)  3) ﻿For each item in the questionnaire, nurses were significantly more satisfied ﻿with CPOE orders than with HW orders (*P* < .0001). |
| Trbovich et al. 2010 [49]  Canada | Controlled observational simulation study (Low), simulated hospital setting | ﻿Smart infusion pump with barcode vs. smart infusion pump (vs. conventional infusion pump) | 504 in total; 168 smart infusion pump with barcode, 168 smart infusion pump and 168 control (simulated tasks performed by 24 nurses acting as both interventions and control) | Medication errors detected by direct observation | ﻿The nurses remedied 60% of wrong drug errors. This rate did not vary as a function of pump type. The nurses remedied wrong patient errors more often when using the barcode pump (88%) than when using the traditional pump (46%) or the smart pump (58%) (Cochran Q=14.36; *P* < 0.05). The number of nurses who remedied wrong dose hard limit errors was higher when using the smart pump (75%) and the barcode pump (79%) than when using the traditional pump (38%) (Cochran Q=12.13;  *P* < .003). There was no difference in remediation of wrong dose soft limit errors across pump types. The nurses’ pump programming was less accurate when mathematical conversions were required. Success rates on secondary infusions were low (55.6%) and did not vary as a function of pump type. |
| White et al. 2010 [50]  Canada | ﻿High-fidelity simulation study (observational, cross-over controlled) (Low),  simulated ambulatory chemotherapy setting | A revised checklist for the verification of ambulatory infusion pump settings (vs. the old checklist) | 140 in total; 70 with revised checklist and 70 with old checklist  (scenarios performed by 10 nurses acting as both intervention and control) | ﻿1) ﻿Errors in pump programming  2) Errors in patient identification  3) Mismatches between order and label  4) Clinical errors | ﻿1) ﻿No significant difference in detection of pump programming errors: 90% with the old checklist (51/60) and 80% with the new checklist (48/60) (*P* > .05). ﻿It was expected that change in the order of items would improve the rate of error detection by eliminating confirmation bias.  2) ﻿Detection of identification errors with the new checklist (80%; 16/20) was significantly higher than with the old checklist (15%; 3/20) (*P* < .01). A specific instruction to check the patient’s identification improved error detection.  3) ﻿Overall, detection of mismatch errors was low, and there was no significant difference between the old checklist (45%; 9/20) and the new checklist (60%; 12/20) (*P* > .05).  4) ﻿Neither checklist helped nurses to identify clinical errors (none of these was detected; 0/30). Thus, the addition of the general reminder to stop and think critically had no impact on error detection (*P* > .05) |
| **Controlled observational review of drug chart, medication orders or patient record** **(n=11)** | | | | | |
| Bailey et al. 2015 [76]  United States | Retrospective controlled observational review of patient record (Low), Pediatric Emergency Department (ED) | Order verification by emergency medicine pharmacist (EPh) present in emergency department (vs. no EPh present, order verification in hospital pharmacy) | 142 in total; 91 intervention and 51 control | 1) Incidence of appropriate antimicrobial selection and dosing  2) Time from order to verification | When EPh present, patients received appropriate first antibiotic 93.4% of the time (vs. 86.3%, *P* = .157), second antibiotic 96.8% of the time (vs. 83.3%, *P* = .023) and appropriate third antibiotic 78.6% of the time (vs. 85,7%,  *P* = .694)  2) Time from order to verification for the first 2 doses was shorter in the EPh group (10.5 min, *P* = .003; 11.4 min,  *P* = .047). |
| Bertsche et al. 2008 [55]  Germany | Prospective controlled observational review of drug chart (Low), ICU | A standard operating procedure (SOP) to prevent frequent and well-documented incompatibilities (vs. before SOP) | 50 in total; 25 intervention and 25 control | Prevalence of  incompatible drug pairs | Patients were given mean ± S.D., 6.7 ± 2.4 different i.v. drugs before and mean ± S.D., 6.8 ± 2.7 after intervention. The frequency of incompatible drug pairs was reduced by the time of intervention from 5.8% (before intervention) to 2.4% (after intervention). Incompatible drug pairs that were governed by the new SOP were reduced from 1.9% to 0.5%. |
| Bertsche et al. 2010 [56]  Germany | Prospective controlled observational review of drug chart (Low), Cardiovascular ICU | Recommendations to avoid Y-site incompatibilities: purging before and after pantoprazole bolus and using 4-lumen instead of 3-lumen catheters (vs. before intervention) | 111 in total; 58 intervention and 53 control | 1) Overall number of patients with at least one incompatible drug pair  2) Number of patients receiving incompatible pantoprazole combinations | 1) Number of patients with incompatibilities was not influenced by the intervention with 36/58 (62.1%) compared to controls with 38/53 (71.7%, *P* = .28).  2) Number of patients receiving incompatible pantoprazole combinations decreased from 15/15 patients receiving pantoprazole (100.0%) in controls to 9/16 (56.2%) in the intervention group (*P* < .01). |
| Boord ym. 2007 [33]  United States | ﻿Retrospective controlled observational review of patient record (Low), surgical intensive care unit (SICU) setting | ﻿Intravenous insulin protocol integrated into CPOE system (vs. manual protocol) | 106 in total; 69 intervention and 37 control (patients on IV insulin for ≥24 hours) | 1) percentage of glucose readings in ideal range of 70–109 mg/dl  2) minutes spent in ideal range of control during the first 5 days of SICU stay  3) ﻿time from first SICU glucose measurement to IV insulin initiation | ﻿1) ﻿The overall percentage of readings 70–109 mg/dl for all 5 measurement days was 29.3% in the control group and 37.7% in the intervention group (a statistically significant difference of 8.4%, *P* = 0.006, permutation analysis). For SICU days 2 and 3, differences of roughly 10%–12% were observed (*P* = .046 and .043). Episodes of severe hypoglycemia (<40 mg/dl) were extremely rare at just 0.2% of all readings for both periods (control, 4/2,178; intervention, 9/4,065 readings).  2) ﻿The mixed model of estimated minutes in ideal range yielded an overall average improvement of 116 minutes, which was statistically significant at *P* = .029. The intervention group spent more minutes per day in the ideal blood glucose range.  3) ﻿Time to initiation was significantly shorter (*P* < .001, log-rank test) in the intervention group than in control group. In the intervention group, IV insulin drips began within the first 12 hours of SICU admission; in the control group, initiation often occurred on the second or third day. |
| Dubrofsky et al. 2016 [51]  Canada | Retrospective controlled observational review of patient record (Low), Acute care setting | Interdisciplinary quality improvement intervention in increasing the dilution of acyclovir before IV administration (vs. before intervention) | 84 in total; 40 intervention and 44 control | 1) Volume (mL) in which each acyclovir dose was delivered  2) Hourly rate of fluid administration  3) Frequency of an increase in hourly hydration rate  4) Incidence of acute kidney injury. | 1) The volume in which each acyclovir dose was administered was significantly higher in the post- intervention group (median 250 mL versus 100 mL,  *P* < .001).  2) The median rate of administration of IV fluid was similar between the 2 groups (100 mL/h for both groups, *p* = 0.19).  3) After the intervention, the rate of infusion of IV fluid was more frequently increased at the time of acyclovir initiation, but the difference was not statistically significant (56% versus 41% of patients, *P* = .17).  4) 7 patients in the pre-intervention group and 8 patients in the post-intervention group met the pre-specified criteria for acute kidney injury, specifically a 30% increase in serum creatinine (18% versus 21%, *P* = .77). |
| Kaplan et al. 2005 [52]  Canada | Prospective controlled observational review of patient record (including a retrospective study to develop intervention) (Low), university hospital setting | Multidisciplinary intervention to improve intravenous proton pump inhibitor (IV PPI) prescribing: education, computerized dose template, pharmacist’s order review, gastroenterology consultation when ordering continuous infusion (vs. before intervention) | 218 in total; 105 intervention (67 for ﻿upper gastrointestinal bleeding (UGIB), 38 for non-UGIB; an additional 30 non-UGIB patients’ IV PPI were substituted to oral PPI by a pharmacist) | 1) Incidence of prescribing errors  2) IV PPI costs  3) Potential confounders (comparison between UGIB and non-UGIB groups) | 1) In UGIB subgroup there was a 26% (95% CI, 10%–42%;  *P* < .0001) reduction and in non-UGIB group 41% (95% CI, 24%–58%; *P* < .0001) reduction in patients without an appropriate indication.  2) There was no statistically significant pharmaceutical cost difference between control and intervention period, with median weekly costs of $4,188 and $4,485, respectively.  3) There was no significant difference in mean age of patients (62 vs. 62), gender proportion (58% male vs. 64% male), total number of hospital admissions (5,739 vs. 5,884), total number of UGIB cases in ED (106 vs. 117), and total number of gastroenterology consultations (286 vs. 331). |
| Lehmann et al. 2006 [34]  United States | Prospective controlled observational review of medication orders (Low),  children’s hospital at an academic medical center | Web-based calculator and decision support system (vs. before intervention: hand-written orders) | 291 in total; 142 intervention (and 20 hand-written orders in intervention period),  129 control  (orders) | 1) Number and type of errors in handwritten and calculator-generated orders  2) Number and type of errors in pharmacy infusion preparation | 1) In control period 27% of all orders contained at least one error vs. 13.6% in intervention period (*P* ≤ .01). Of all handwritten orders (intervention and control period) 55% contained at least one error, compared with 6% of calculator-generated orders (intervention). In handwritten orders, the most frequent error types were wrong concentration (10.1%), wrong dose (9.4%) and wrong use of the Rule of Six (7.4%). There was a dramatic reduction of the most frequent error types (wrong concentration, 0%; wrong dose, 0%; wrong calculation, 0%) in calculator-generated orders and they were associated with fewer pharmacy interventions.  2) In 118 sequential pharmacy infusion preparations over 4 weeks, no errors were observed. |
| Manjaly et al. 2012 [60]  United Kingdom | Retrospective controlled observational review of drug chart, ﻿case notes and hospital antibiotic database (Low); university hospital setting | Electronic dose calculator for Gentamicin prescriptions in 2009 and 2010 (vs. manual calculations in 2008) | 90 in total;  32 intervention group 1 (2009; 20 non-obese, 12 obese), 27 intervention group 2 (2010; 17 non-obese, 10 obese), 31 control (2008; 17 non-obese, 14 obese) | 1) Accuracy of dose and frequency prescription of Gentamicin  2) Time frame for measurement of serum Gentamicin levels | 1) In all groups, 71–88% of non-obese patients were given only correct doses. In total, 22% of non-obese patients had a dose prescribed incorrectly. 43% of obese patients in control group received incorrect doses compared to 42% in intervention group 1 and 20% in intervention group 2. In control group 12.2% (13/107; 9 in obese patients) of prescriptions were at the incorrect frequency, in intervention group 1 3.8% (4/106; 3 in obese patients) and in intervention group 2 4% (3/75; 2 in obese patients).  2) Median time for drug level samples to reach pathology in all measurement periods was 70–91 min, but differences were not statistically significant (*P* = .274). On average, doses could be administered within 2.5 h of a blood sample being taken. |
| Muzyk ym. 2012 [35]  United States | Retrospective controlled observational review of patient record (Low), hospital ﻿general medical unit | ﻿CPOE set for IV haloperidol treatment monitoring: monitoring parameters, maximum and cumulative doses, identification or mitigation of risk factors for QTc prolongation (vs. before intervention) | ﻿151 in total; 67 intervention and 84 control | 1) ﻿24 h cumulative dose of intravenous haloperidol  2) ﻿incidence of baseline ECG  3) incidence of follow-up ECG within 24 h of IV  4) magnesium and potassium value assessment at the time of IV haloperidol administration | 1) ﻿Patients in the intervention group were more likely to receive a 24 h cumulative dose of IV haloperidol <2mg (﻿47.8% before intervention vs. 64.3% after, *P* < .048)  2) Patients in the intervention group were more likely to have a baseline ECG (65.5% before intervention vs. 80.6% after, *P* = .045)  3) Patients in the intervention group were more likely to have a follow-up ECG within 24 hours of intravenous haloperidol administration (25.2% of potential monitoring opportunities before intervention vs. 58.5% after)  4) Patients in the intervention group were more likely to have a magnesium value assessed at the time of intravenous haloperidol administration (51.2% before intervention vs. 74.6% after, *P* = .004). More patients were lacking a potassium value at the time of IV haloperidol administration in the intervention group (6.0% before intervention vs. 13.4% after, not statistically significant). |
| Nuckols et al. 2008 [36]  United States | Retrospective observational review of medical record (Low), intensive care unit setting | Smart infusion pumps (vs. conventional infusion pumps) | ﻿4,604 in total (number of patients in intervention and control group not reported; 20,559 bed-days in total) | ﻿1) preventable IV-ADEs matching smart-pump features  2) error types involved in preventable IV-ADEs | ﻿1) Of 100 preventable IV-ADEs identified, 4 involved errors matching smart-pump features. 2 occurred before and 2 after smart-pump implementation. However, given that there were few preventable IV-ADEs, statistical power was severely inadequate. The conventional-pump rate was 4.78 per 1,000 patient-days and the smart-pump rate was 4.95 (adjusted mean difference 0.04 per 1,000 patient-days, *P* = .96).  2) Overall, 29% of preventable IV-ADEs involved overdoses; 37%, failures to monitor for potential problems; and 45%, failures to intervene when problems appeared. Error descriptions suggested that expanding smart pumps’ capabilities might enable them to prevent more IV-ADEs. |
| Pell ym. 2014 [37]  United States | Controlled observational review of patient record (Low),  tertiary care hospital setting | ﻿Focused computerized alert at the time of medication order entry triggered only for patients with prolonged QTc to decrease unsafe use of IV haloperidol in this high-risk patient population (vs. ﻿no alert) | 153 in total; 87 intervention (average of 10.9/month) and 66 control (average of 8.3/month)  (orders for IV haloperidol in patients with a QTc >500 ms; alerts fired to prescriber in the intervention period and alerts fired to researchers in the control period) | 1) The proportion of inappropriate prescribing of all alerts  2) Inappropriate IV haloperidol prescribing  3) Actions taken after the alert (intervention period only)  4) Overall rate of IV haloperidol prescriptions (control period only to determine how often a non-customized alert would have fired) | ﻿1) ﻿In the control period, 50.0% of alerts were in patients receiving end of life care or patients with a prolonged QRS and 50.0% were inappropriate prescribing. In the intervention period, ﻿66.7% of alerts were in patients receiving end of life care or patients with a prolonged QRS and 33.3% were inappropriate prescribing.  ﻿2) A decrease in the rate of completed inappropriate haloperidol prescriptions was observed from an average of 4.1/month in control group to 1.5/month after intervention (*P* = .00025). The proportion of patients administered inappropriate haloperidol dropped from 50% to 14%.  3) ﻿The IV haloperidol order was abandoned in 40 (46.0%) patients with a QTc >500 ms on whom the alert fired. Of these 40, 18 (45.0%) would have been inappropriate prescribing and 22 (55.0%) were on patients with prolonged QRS or end of life care. During the intervention period, 12 patients were prescribed what was considered inappropriate IV haloperidol despite the alert.  4) Overall rate of IV haloperidol prescriptions in control period was 1,124. ﻿Without the specific alert the amount of alerts fired would have been much higher (8.3/month vs. 140.5/month). |
| **Controlled observational analyses of medication error or adverse drug event data (n=3)** | | | | | |
| Guérin et al. 2015 [53]  Canada | Retrospective observational analysis of medication error data (Low)  women’s and pediatric hospital setting | Smart infusion pumps (vs. conventional infusion pumps) | 1,045 (smart infusion pumps implemented in 500-bed hospital; two one-year intervention periods and one one-year control period) | Drug-related accidents and incidents (detected by self-reporting) | ﻿﻿No risk reduction associated with the implementation of smart pumps was observed. A total of 2,911 accident and incident events related to medications, devices, and equipment were self-reported by clinical staff in the control period, 3,523 in first year of intervention, and 2,788 in the second year of intervention. The intravenous accidents and incidents increased from 1,432 in control period to 1,834 in first year of intervention and decreased to 1,389 in second year of intervention. |
| Larsen et al. 2005 [38]  United States | Observational analysis of medication error data (Low), pediatric hospital setting | Smart infusion pumps utilizing drug library, standard concentrations and ﻿human-engineered medication labels (vs. before intervention) | 24,508 in total; 12,399 intervention and 12,109 control (continuous drug infusions) | 1) medication errors (detected by self-reporting)  2) use of standard concentrations | ﻿1) The number of reported errors dropped by 73%, giving an absolute risk reduction of 3.1 to 0.8 per 1,000 doses. Pharmacy preparation errors decreased from 0.66 to 0.16 per 1000 doses; the number of 10-fold errors in dosage decreased from 0.41 to 0.08 per 1,000 doses. ﻿The hospital-wide incident-reporting rate was identical for control and intervention periods (0.03 incidents reported per inpatient day).  2) 87% of the continuous medication infusions in the NICU and >99% in other areas of the hospital used standard concentrations. |
| Reece et al. 2016 [40]  United States | ﻿Observational analysis of medication error data (Low),  oncology ambulatory care pharmacy setting | ﻿Gravimetric IV workflow software system ﻿using barcode verification, real-time alerts and automatic error reporting (vs. conventional volumetric preparation method with self-reporting) | 66,880 in total; 15,843 intervention and 51,037 control (compounded IV doses) | 1) medication errors (﻿control group: self-reported errors detected in visual inspection; intervention group: error reports generated by the workflow software system)  2) time to task completion | ﻿1) More errors were identified in the intervention group (1,126 errors/15,843 doses; 7.1%) than in control group (49 errors/51,037 doses; 0.096%). ﻿Barcode scanning detected 26% of the total errors, gravimetric weighing detected 71% of errors, and 3% errors were detected at vial reconstitution.  2) ﻿Technician production time decreased by 34% (from 9.2 ± 6.9 min to 6.0 ± 4.3 min) by using the workflow software system, and pharmacist checking time decreased by 37% (from 3.17 min to 2.00 ± 0.78 min). |
| **Observational study combining multiple methods** **(n=4)** | | | | | |
| Barras ym. 2014 [67]  Australia | Observational multi-factorial approach (assessment of current state, development and implementation of safety intervention; includes comparison) (Low), hematology hospital setting | Safety intervention in intravenous potassium use: a prescribing and monitoring form, premixed bags instead of concentrated ampoules, double- checks, labelling of infusion lines, clinician training and guidelines, “smart pump” software (vs. before intervention) | Not reported (12 months follow-up periods before and after intervention) | 1) The number of ampoules and premixed infusions dispensed to the ward  2) The number of reported clinical incidents in 12 months | 1) A total of 10,100 ampoules of concentrated potassium were used (841 per month) in the 12 months before intervention. There were no ampoules used in the 12 months following the system changes.  2) There were significantly fewer incidents in the post-implementation period compared to the pre-implementation period (23 vs. 9, *P* < .001). |
| Vardi ym. 2007 [70]  Israel | ﻿Controlled observational  study combining multiple methods  (analysis of self-reported errors, errors detected by drug chart review, simulation study) (Low), pediatric critical care setting | CPOE and decision support system generated ﻿orders for pediatric resuscitations (vs. handwritten orders) | 60,094 in total; 46,970 intervention and ﻿13,124 control ﻿(patients; time to task completion was measured for preparation of 128 simulated conventional forms and 128 CPOE forms) | 1) medication errors (control period: self-reported errors; intervention period: errors detected in drug chart review)  2) ﻿time to task completion (preparation of the resuscitation  drug forms) | ﻿1) There were three reported incidents of errors in the control period, which represent errors that escaped the triple check by three independent staff members. There were no errors in the intervention period (100% error reduction).  2) Time to completion of drug forms dropped from 14 min 42 s to 2 min 14 s (*P* < .001). |
| Cour et al. 2013 [69]  France | Prospective quasi-experimental study (Low), intensive care unit setting | ﻿Automated changeover of vasoactive drug infusion pumps  (vs. manual changeover of vasoactive drug infusion pumps) | ﻿133 in total; 70 intervention and 63 control (1,329 relays in total; 648 intervention and 681 control) | 1) Hemodynamic incidents (﻿variation of MAP >15 mmHg or HR >15 bpm) related to changeover of vasoactive drug infusion pumps (recorded by monitoring device)  2) workload related to changeover of vasoactive drug infusion pumps (self-reported) | ﻿1) Decrease in incidents (137 (20%) before intervention vs. 73 (11%) after, *P* < .001). Automated relays were independently associated with a 49% risk reduction of incidents induced by changeover of vasoactive drug infusion pumps (adjusted OR=0.51, 95% confidence interval 0.34–0.77, *P* = .001).  2) Time dedicated to the relays and the number of interruptions in care to manage CVIP were significantly reduced with automated relays vs. manual relays  (*P* = .001). |
| Evley ym. 2010 [61]  United Kingdom | Qualitative observational study combining direct observation and focus group interviews (Low), hospital anesthesia department setting | Electronic barcode confirmation of drugs (vs. double-check performed by second person) | ﻿Not applicable  (7 observation sites for 3 months in total, 2 intervention and 5 control; 4 focus groups) | ﻿Feasibility of the used methods (benefits, disadvantages and practicalities) | ﻿Both methods were perceived to contribute to the prevention of drug errors. For the two-person confirmation to be carried out correctly, there should be no distraction or time pressure. The main limitation to the feasibility was that the continuous presence of the second person was not always possible. The process also met with resistance from the staff at some pilot sites. Electronic confirmation was always feasible, as it did not require the presence of a second person. It was found to be intuitive to the anesthetist’s current working practice. However, there were some practical issues related to the introduction of new technology and an initial learning curve. |
| **Controlled observational study involving analyses of infusion concentrations (n=2)** | | | | | |
| Campino et al. 2016 [64]  Spain | Prospective controlled observation study (Low), ten Neonatal Intensive Care Units (NICUs) and hospital pharmacy | Standard concentrations, preparation protocols and educational program (vs. before intervention) | 876 in total; 372 intervention and 504 control (samples) | 1) Calculation errors in preparation  2) Accuracy errors in preparation | 1) In NICUs, 1.35% of samples registered calculation errors in pre-intervention phase; no calculation errors were registered in hospital pharmacy samples. In post-intervention phase, no calculation errors were registered in either group.  2) Pre-intervention: ﻿In NICUs, a statistically significant  (*P* < .001) lack of accuracy was detected in 243/444 samples (54.7%). In ﻿HPS, a lack of accuracy was detected in 23/60 samples (38.3%). ﻿In the post-intervention phase the difference between the overall accuracy error rate in NICUs and the HPS was statistically significant (*P* = .017 Chi2). ﻿The difference between the overall accuracy error rate in NICUs and the HPS was not statistically significant (*P* = .225 Chi2). Accuracy error rate after the intervention phase decreases significantly both in NICUs (54.7 vs. 23%; *P* < .001 Chi2) and HPS (38.3 vs. 14.6%; *P* < .010 Chi2). |
| Dehmel et al. 2011 [57]  Germany | Prospective controlled observation study (Low), ICU and hospital pharmacy | Pharmacy-based automated infusion solution production (vs. manual ward-based production) | 200 in total; 100 intervention and 100 control (samples) | 1) Concentration of solution  2) Deviations from stated concentrations | 1) The concentration of 53 (53%) ward-prepared and 16 (16%) pharmacy-produced solutions deviated at least 5% above or below the intended concentration. The mean concentration of manually prepared solutions was 97.2% (SD 12.7%, range 45–129%) and of machine-made solutions was 101.1% (SD 4.3%, range 90–114%) of the target concentration (*P* < .01).  2) Deviations of more than 10% were measured in 22 (22%) of manually prepared and 5 (5%) of pharmacy-prepared samples. 15 (15%) of the manually prepared solutions deviated by more than 15% of the stated concentration, the maximum deviation being 55% below the declared concentration. The maximum deviation in machine-made solutions was 14%. |
| **LOW EVIDENCE QUALITY: UNCONTROLLED OBSERVATIONAL STUDIES (n=7): observational analysis of pump programming or preparation error data (n=5), descriptive validation study using Delphi method (n=1), experimental study (n=1)** | | | | | |
| Chanes et al. 2012 [68]  United States and Brazil | Descriptive validation study using Delphi method (Low), pediatric oncology setting | Two algorithms to prevent extravasation in children receiving chemotherapy through peripheral line (no comparison) | 14  (Delphi panelists) | Consensus equal to or higher than 80% | Consensus was reached as the agreement between both instruments ranged from 92.8% to 99.0%. |
| Deng et al. 2016 [41]  United States | Retrospective observational analysis of preparation error data (Low),  pediatric hospital pharmacy setting | ﻿Automated workflow management system for compounding of IV medications (no comparison) | ﻿421,730 (compounded IV doses) | 1) ﻿Rate of medication errors (﻿error reports generated by the automated IV compounding workflow management system)  2) ﻿Factors associated with IV compounding errors | ﻿1) 3,101 documented errors (an overall error rate of 0.74%). The automated system intercepted 72.27% of errors, mainly those containing an incorrect drug or diluent. 27.73% of IV compounding errors, primarily dose preparation in the wrong volume (21.51%) or damage to the final product (0.93%), were identified during final inspection by a pharmacist.  2) The logistic regression model showed that four factors were significantly (*P* < .05) associated with an increased risk of errors: dose preparation during the morning shift (relative risk [RR], 1.84; 95% CI, 1.68–2.02) or on a Sunday (RR, 1.28; 95% CI, 1.11–1.47), preparation of doses for use in critical care units (RR, 1.17; 95% CI, 1.07–1.28), and technician versus pharmacist compounding (RR, 1.17; 95% CI, 1.04–1.32). |
| Evans et al. 2010 [42]  United States | Observational analysis of pump programming error data (Low), university hospital shock/trauma intensive care unit setting | ﻿Smart system connected to the electronic medical records to reduce adverse drug events by enhanced notification of infusion pump programming errors (no comparison) | ﻿3,865 (a total of 13,648 patient days; ﻿25,040 drug doses delivered via an infusion pump) | 1) Potential prevented medication errors (detected from electronic medical record ﻿alert log data)  2) Outcomes and potential harm caused by errors | 1) ﻿970 doses (4%, 1.4 alerts per day) generated an alert due to pump settings that were outside the acceptable ranges.  2) 137 alerts (14% of alerts, 10/1,000 patient days) prevented potential patient harm. ﻿All of them were the result of obvious infusion pump programming errors and for all but two, the pump settings were fixed at the time of the alerts. |
| Kaakeh et al. 2008 [44]  United States | Experimental study of the sensitivity and specificity of a laboratory device (Low), pediatric hospital pharmacy setting | Tabletop enhanced photoemission spectroscopy (EPS) for verification of compounded IV admixtures (no control) | 700 in total; no control (samples; 7 medications, 10 x 10 sample sets for each medication) | 1) Accuracy of concentration verification (validity of dilutions)  2) Accuracy of drug identification (cross-testing) | 1) The EPS device detected errors departing from the targeted concentration by 20% or more with a sensitivity of at least 95%. For all medications, the instrument correctly did not provide validation of concentrations 5- or 10-fold higher or > 50% lower than the targeted concentration. For samples at targeted concentrations, the machine correctly validated the samples in 100% of cases. All the drugs at 90% and 110% of targeted concentrations were validated 80–100% of the time.  2) 20% of lorazepam samples at the standard concentration of 1 mg/mL were validated as gentamicin. This problem was communicated to the manufacturer so that it could adjust the signature, and the problem was corrected. |
| Kastrup et al. 2012 [58]  Germany | Retrospective observational analysis of pump programming error data (Low), intensive care unit setting | Smart infusion pumps and drug library (no comparison) | 7,884 (patient treatment days; 133,601 infusion starts) | 1) Compliance in drug library use  2) Potential prevented medication errors (detected from smart-pump  ﻿alert log data) | ﻿1) The drug library with the features of the dose rate was used in 92.8% of the syringe pump starts. In 1.5% of the starts a manual dosing mode without the use of the drug library was employed and in 5.7% of the starts the mode ‘mL/h’, without any calculation features, was used.  2) The user was alerted to potentially harmful overdosing in 717 cases and in 66 cases the pumps were reprogrammed after the alert. |
| Manrique-Rodríguez et al. 2013 [66]  Spain | Prospective observational analysis of pump programming error data (Low), pediatric intensive care unit setting | Smart infusion pumps and drug library (no comparison) | ﻿624,252 (started infusions) | 1) Compliance in drug library use  2) ﻿The number of prevented programming errors (based on the alerts detected by the safety software)  3) Potential severity of errors | 1) Of all started infusions, 78% (n=486,875 were programmed through the drug library.  2) ﻿ 92 programming errors, of which about 97% of the errors resulted from user programming of doses or infusion rates above the hard limits defined in the smart pump drug library. The percentage of infusions reprogrammed after a related soft-limit alert was 30% (i.e., 70% of alerts were overridden).  3) The potential consequences of the intercepted errors were considered to be of moderate, serious, or catastrophic severity in 49% (45/92) of cases. |
| Williams et al. 2006 [45]  United States | Observational analysis of pump programming error data (Low), tertiary care hospital setting | Smart pumps with drug library (including standard concentrations and dose limits) (no comparison) | ﻿5,139 (alerts generated by 426 infusion pumps over 9 months’ time) | 1) Number of averted medication errors (detected from infusion pump alert log)  2) Number of averted overdoses | ﻿1) ﻿A total of 245 alerts resulted in a programming change or canceled infusion and were classified as averted errors.  2) Of 166 averted overdoses, 100 (60.2%) involved high-risk medications, and 105 (63.3%) were medications unlikely to be detected in the event of overdose. IV Medication Harm Index scores ranged from 3.5 to 13 (mean, 8.2; range, 3.5 to 14), and 33 (19.9%) averted overdoses scored 11 or higher. Sorted by frequency only, propofol, heparin, and dopamine accounted for more than half of all averted overdoses; however, IV Medication Harm Index analysis showed that heparin and propofol accounted for almost three-quarters of the highest-risk averted overdoses. |
